# Online Collision-Induced Unfolding of Therapeutic Monoclonal Antibody Glyco-Variants through Direct Hyphenation of Cation Exchange Chromatography with Native Ion Mobility–Mass Spectrometry

**DOI:** 10.1021/acs.analchem.2c03163

**Published:** 2023-02-15

**Authors:** Guusje van Schaick, Elena Domínguez-Vega, Jérôme Castel, Manfred Wuhrer, Oscar Hernandez-Alba, Sarah Cianférani

**Affiliations:** † Center for Proteomics and Metabolomics, Leiden University Medical Center, Albinusdreef 2, 2333 ZA Leiden, The Netherlands; ‡ Laboratoire de Spectrométrie de Masse BioOrganique, IPHC UMR 7178, Université de Strasbourg, CNRS, Strasbourg 67087, France; § Infrastructure Nationale de Protéomique ProFI, FR2048 CNRS CEA, Strasbourg 67087, France

## Abstract

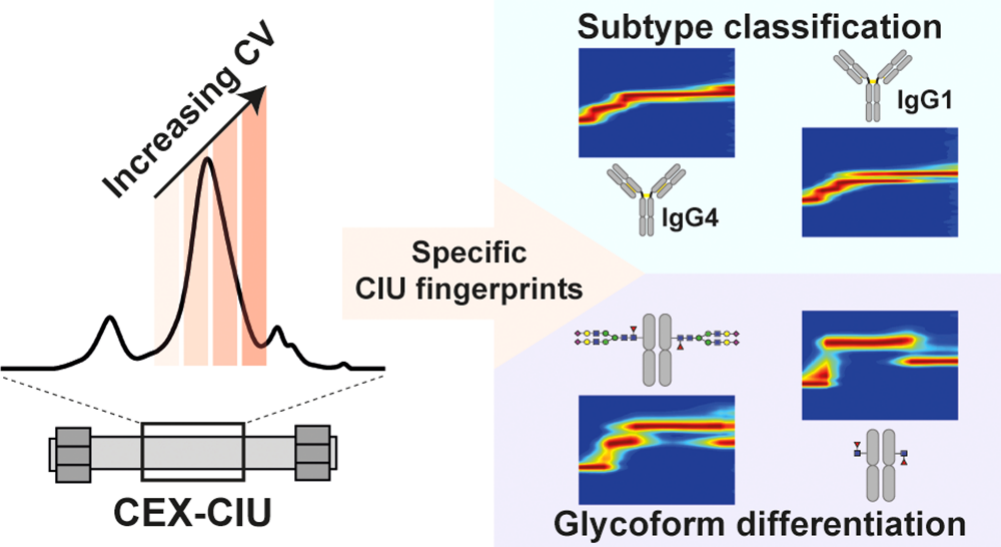

Post-translational modifications (PTMs) not only substantially increase structural heterogeneity of proteins but can also alter the conformation or even biological functions. Monitoring of these PTMs is particularly important for therapeutic products, including monoclonal antibodies (mAbs), since their efficacy and safety may depend on the PTM profile. Innovative analytical strategies should be developed to map these PTMs as well as explore possible induced conformational changes. Cation-exchange chromatography (CEX) coupled with native mass spectrometry has already emerged as a valuable asset for the characterization of mAb charge variants. Nevertheless, questions regarding protein conformation cannot be explored using this approach. Thus, we have combined CEX separation with collision-induced unfolding (CIU) experiments to monitor the unfolding pattern of separated mAbs and thereby pick up subtle conformational differences without impairing the CEX resolution. Using this novel strategy, only four CEX–CIU runs had to be recorded for a complete CIU fingerprint either at the intact mAb level or after enzymatic digestion at the mAb subunit level. As a proof of concept, CEX–CIU was first used for an isobaric mAb mixture to highlight the possibility to acquire individual CIU fingerprints of CEX-separated species without compromising CEX separation performances. CEX–CIU was next successfully applied to conformational characterization of mAb glyco-variants, in order to derive glycoform-specific information on the gas-phase unfolding, and CIU patterns of Fc fragments, revealing increased resistance of sialylated glycoforms against gas-phase unfolding. Altogether, we demonstrated the possibilities and benefits of combining CEX with CIU for in-depth characterization of mAb glycoforms, paving the way for linking conformational changes and resistance to gas-phase unfolding charge variants.

Monoclonal antibodies (mAbs) have played a dominant role in the treatment of various disorders,^1,2^ and this class of human therapeutics is still rapidly expanding. Currently, most therapeutic mAbs are IgG1 subclass, but other IgG subclass formats are also being produced (IgG2 or IgG4).^3^ Besides a variation in subclass, therapeutic mAbs can contain a plethora of different post-translational modifications (PTMs), including lysine clipping, pyroglutamate formation, glycosylation, oxidation, and deamidation.^4,5^ Changes in the PTM profile may influence the conformation and thereby impact biological functions (i.e., efficacy and safety of therapeutic proteins). For instance, mAb charge variants, such as deamidated species and proteoforms with isomerization of asparagine residues, may result in decreased binding affinity and potency.^6^ Novel strategies are needed to fully characterize charge-related structural changes to ensure efficacious and safe therapeutic products.

Over the last decades, cation-exchange chromatography (CEX) coupled with ultraviolet (UV) detection has found its way into quality control as a reference technique to monitor charge variant profiles of mAb-based therapeutic products.^7,8^ Nevertheless, these robust and routinely used methods often lack the ability to perform in-depth structural characterization without time-consuming fraction collection followed by offline mass spectrometry (MS). Fortunately, recent advances dealing with interfacing CEX and native MS (nMS) enabled the development of methodologies providing online separation, identification, and characterization of charge variants.^9^ Most importantly, the replacement of traditional non-volatile salt gradients with low ionic strength pH gradients using (MS-compatible) ammonium-based mobile phases boosted the application of CEX–nMS for charge variant analysis.^5,10,11^ While these pH gradients already result in sufficient separation efficiency for most proteins, proteins with high isoelectric points (pIs) or heterogeneous proteins consisting of many proteoforms may benefit from a pH gradient accompanied by a (minor) increase in the salt concentration, a so-called salt-mediated pH gradient.^10,12^ Especially, the latter enabled online identification of mAb proteoforms with altered pI, such as proteoforms with differences in glycosylation, deamidation, and isomerization.^7,13,14^ Currently. CEX–nMS applications focus largely on structural characterization of charge variants, while their effect on the protein conformation or gas-phase unfolding pattern is not explored.

To decipher the conformational landscape of mAbs, ion mobility (IM)-based technologies are particularly suitable.^15^ The availability of traveling wave IM spectrometry in commercial MS instruments opened many possibilities to study global protein conformation in the gas phase.^16^ Unfortunately, IM–MS measurements often have low resolving power for species with related conformations. As a consequence, the collision cross sections (CCSs) obtained from these measurements are often not very informative for intact mAb analysis.^3,17^ To circumvent this poor resolution as well as gain deeper insights into the gas-phase behavior after activation, IM-based collision-induced unfolding (CIU) has been proposed as a suitable alternative. So far, CIU has been employed to investigate different properties and modifications of mAbs^3,17−19^ and antibody–drug conjugates.^20,21^ While these classical CIU approaches face various practical challenges preventing routine use, including manual buffer exchange and time-consuming data acquisition process, online coupling of size exclusion chromatography (SEC) with CIU allowed automation of this workflow tackling these challenges.^22^

Here, we present an innovative online CEX–CIU method for the in-depth characterization of different mAb populations in their native state in a fast and straightforward manner. Using this approach, mAb populations were separated according to their pIs, and their specific unfolding patterns were acquired by increasing the collision voltage (CV) in the trap cell prior to IM separation during elution of the selected mAb population. This analytical strategy was developed to record the CIU fingerprints of intact or IdeS-digested mAbs showing the capability of this technique to generate CIU fingerprints of CEX-separated protein populations. Subsequently, CEX–CIU hyphenation was tailored to decipher conformational differences of an equimolar mixture of three nearly isobaric mAbs. Finally, the potential of CEX–CIU for charge variant analysis is illustrated by studying gas-phase unfolding resistance of different remodeled glycan-variants of a reference mAb.

## Experimental Section

### Materials

Eculizumab (Soliris, Alexion), trastuzumab (Herceptin, Roche), and pembrolizumab (Keytruda, Merck) were obtained from their manufacturers. Glycan remodeling was performed by treatment of trastuzumab with TransGLYCIT (Genovis, Lund, Sweden) according to the producer specifications with and without the presence of a fucosidase. For middle-level analysis, enzymatic digestion was performed by incubation of one unit of the IdeS enzyme (FabRICATOR, Genovis, Lund, Sweden) per microgram of mAb at 37 °C for 60 min. Prior to further analysis, the samples were buffer exchanged to 50 mM ammonium acetate using 10 kDa Vivaspin 500 filters (Sartorius, Göttingen, Germany) with an end concentration of 2 mg/mL.

### CEX Separation

The CEX measurements were performed using an Acquity UPLC H-class system (Waters, Wilmslow, UK) equipped with a quaternary solvent manager, a sample manager operated at 10 °C, a column oven, and a TUV detector. A BioResolve SCX column (2.1 × 100 mm, 3 μm) was used from Waters. The mobile phases were composed of 50 mM ammonium acetate at pH 5.0 (A) and 160 mM ammonium acetate at pH 8.6 (B). For intact trastuzumab, a linear gradient from 50 to 70% B in 10 min was applied. The middle-level trastuzumab separation was achieved by a linear gradient from 30 to 70% B in 10 min. The mixture of mAbs was separated by first maintaining 0% B for 1 min followed by a linear gradient from 30 to 70% B in 9 min. All CEX methods contained a column cleaning step at 100% B for 3 min and a re-equilibration step at 0% B for 7 min. The injected amount of intact mAbs was 20 μg and that of middle-level mAbs was 12 μg. The flow rate was 0.1 mL/min, and the UV wavelengths were 280 and 214 nm.

### CEX–ESI–CIU Experiments

CEX was coupled online with a Synapt G2 HDMS mass spectrometer (Waters) operated in positive mode with a capillary voltage of 3 kV and a sample cone voltage of 180 V. The backing pressure of the Z-Spray source was increased to 6 mbar. Desolvation and source temperatures were set to 450 and 90 °C, respectively. Desolvation and cone gas flow rates were 750 and 60 L/h, respectively. The cone voltage of the Synapt G2 was lowered to 80 V (for intact analysis) or 60 V (for middle-level analysis). The argon flow rate was set to 2 mL/min. Prior to the IM cell, ions were focused in the helium cell (120 mL/min). In the IM cell, the N_2_ flow of 60 mL/min was applied, and the wave height and velocity were 40 V and 800 m/s. Other settings were similar as mentioned for the CEX–nMS analysis. The *m/z* range was from 1000 to 10,000. External calibration was performed using cesium iodide (2 mg/mL in 50% isopropanol).

For the CEX–CIU experiments, the CV in the trap cell was stepwise increased from 0 to 150 V (in steps of 10 V). One complete CIU fingerprint was obtained in four CEX runs, where each CEX run contained four IM–MS functions per selected species (0–30, 40–70, 80–110, and 120–150 V). For each of these voltage steps, the number of scans and scan time were 4 and 0.2 min, respectively. The start and end times of the functions were adjusted to the retention time of the species of interest, and between the functions during elution, a window of 0.5 s was kept ensuring effective application and stable CVs.

### ESI–CIU Experiments

Intact or middle-level trastuzumab was infused in an electrospray ionization (ESI) source using a syringe pump (KD Scientific, Holliston, MA, USA) with a 250 μL syringe (Hamilton, Bonaduz, Switzerland) at a rate of 3 μL/min. The parameters of the Synapt G2 were as described for CEX–CIU experiments. The CV in the trap cell was manually increased from 0 to 150 V in steps of 10 V, and from each CV, 0.5 min was acquired.

### Data Analysis

The CEX–IM–nMS data were processed with MassLynx (v4.1), and data from CIU experiments were analyzed and visualized using CIUSuite 2 (v2.2)^23^ or ORIGAMI^ANALYSE^ (v1.2.1.6).^24^ The chromatographic resolution was calculated with *R*_s_ = 1.18 × ((*t*_R2_ – *t*_R1_)/(*W*_FWHM1_ + *W*_FWHM2_)), where *t*_r_ is the retention time (min), and *W*_FWHM_ is the peak width at half-height (min). The drift times obtained with CEX–IM–nMS were converted into CCS values using external calibrants, including β-lactoglobulin and concanavalin A for the middle level and concanavalin A, alcohol dehydrogenase, and pyruvate kinase for the intact level.^25^ Arrival time distributions (ATDs) were smoothed with the Savitzky–Golay algorithm with a window length of 5 and polynomial order of 2. The data were interpolated with a factor of 2 on the CV axis. For all CIU analyses, averaged and differential plots, including root-mean-square deviation (RMSD) values, were obtained. Feature detection and CIU50 analysis were performed using standard mode, where the minimum feature length was 2 steps, the feature allowed width was 0.75 ms, no CV gap length within a feature was allowed, drift time spectrum was the centroid at the maximum value for each CV, and transition region padding was 15 V. The univariate feature selection (UFS) plots were generated to highlight diagnostic voltages [i.e., high −log10(*p*-value) scores] to classify mAb subclasses.

## Results and Discussion

### CEX for mAb Charge Variant Separation at Intact and Middle Levels

**Figure 1:**
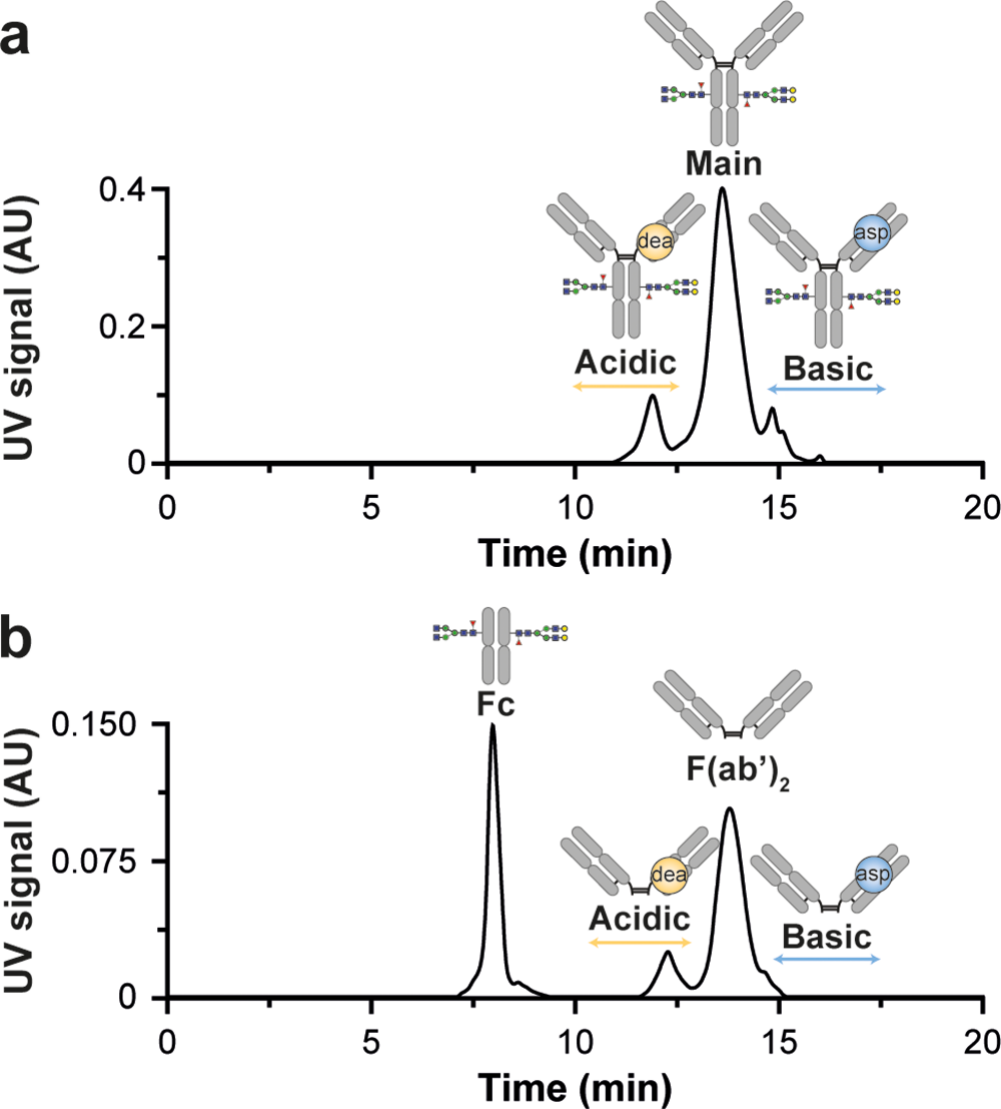
CEX–UV chromatograms of intact trastuzumab (a) and middle-level analysis of trastuzumab (b). For intact trastuzumab, acidic (deamidated proteoforms) and basic species (IsoAsp variants) were separated. The middle-level analysis resulted in two main peaks corresponding to the Fc and F(ab′)_2_ fragments. Similar charge variants as for intact trastuzumab were observed on the F(ab′)_2_ part.

According to the separation capabilities of CEX for therapeutic mAbs, we implemented two different CEX–nMS methods to monitor charge variants of a reference mAb (trastuzumab) at both the intact and middle levels. Most efficient separation of charge variants of trastuzumab was achieved using a salt-mediated pH gradient with mobile phases composed of 50 mM ammonium acetate at pH 5.0 (A) and 160 mM ammonium acetate at pH 8.6 (B). Applying these mobile phases with a gradient from 50 to 70% B, we resolved acidic and basic species from the main form of intact trastuzumab (Figure 1a) with similar separation efficiencies as previously reported.^10,11^ The main form of trastuzumab eluted at 13.6 min with a full width at half-maximum (FWHM) of 0.77 min. Besides this form, additional acidic and basic variants were separated, which could be assigned based on previous (bottom-up) data.^10,11,13,26^ The acidic species, including deamidated variants, eluted at 11.9 min and were baseline resolved from the main peak (resolution of 1.6). Basic charge variants due to isomerization of Asp residues eluted later at 14.9 min (resolution of 1.2) (Figure 1a; Table S1).

Although analysis of intact mAbs can be very informative, CEX–nMS on mAb subunits upon IdeS digestion^27^ (i.e., cleavage of mAb below the hinge region) allowed localization of charge variants at the subunit level along with more precise mass measurements facilitating PTM identification^17^ (Figure 1b). The CEX separation of the Fc and F(ab′)_2_ fragments was accomplished using the same mobile phases with a gradient from 30 to 70% B (Figure 1b; Table S1). While the Fc fragment eluted as a single peak at 8.0 min, multiple peaks were observed for the F(ab′)_2_ domain. Besides the main F(ab′)_2_ peak at 13.9 min, also acidic and basic species were separated at 12.3 and 14.8 min, respectively. The latter indicated that the deamidation and isomerization occur on the F(ab′)_2_ domain, which is also consistent with published data.^26,28^ The peak width of the Fc peak was smaller, whereas the F(ab′)_2_ peak was very similar to intact trastuzumab. This is in line with expectations since low ionic strength mobile phases have been described to focus on the chromatographic peaks, while higher ionic strength results in broader peaks.^8^ In our case, the pH gradient starts at low ionic strength (50 mM) and increases in ionic strength with time.

### Coupling of CEX and CIU

**Figure 2:**
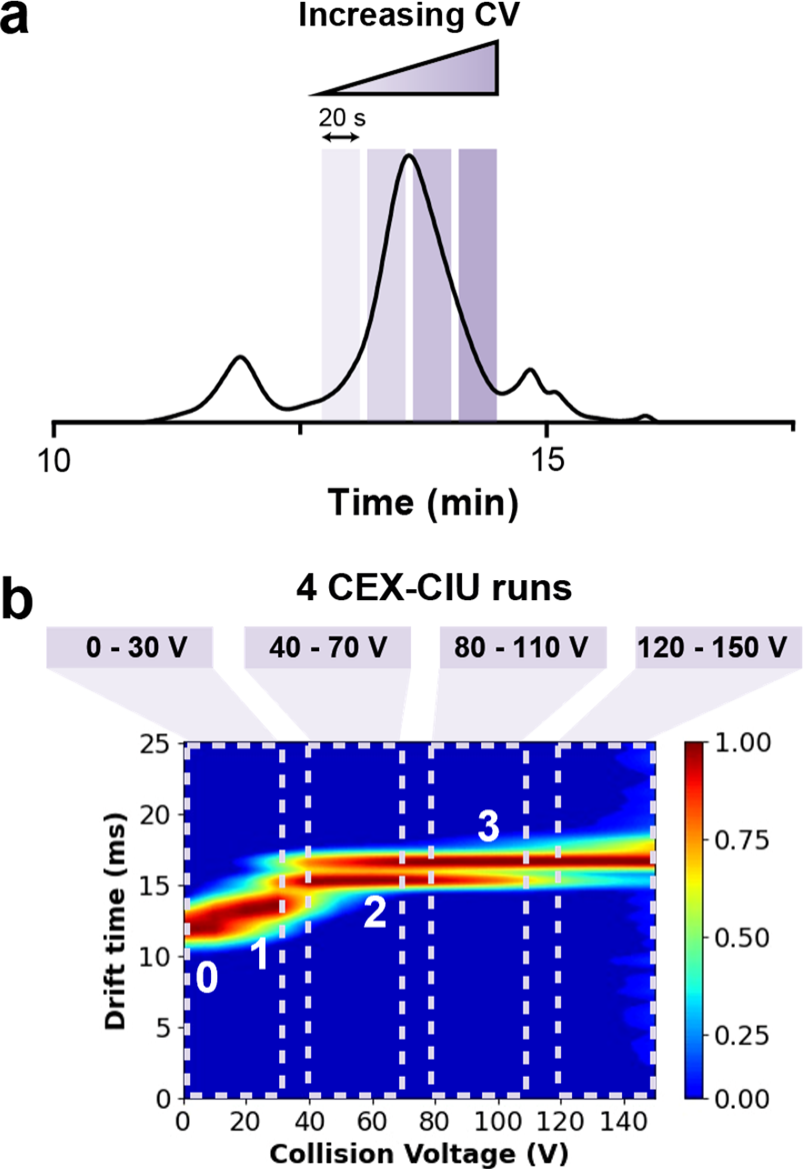
CEX–CIU for mAb analysis. (a) CEX–UV chromatogram of intact trastuzumab showing the increase in CV to obtain the CIU fingerprint. During elution of the selected species, the CV is increased in four steps, where each CV is maintained for 0.2 min. (b) CEX–CIU fingerprint of the 27+ charge state of the trastuzumab peak indicated in the chromatogram. In total, four measurements were required to obtain a fingerprint from 0 to 150 V. The conformational states are labeled with numbers from 0 to 3. The replicate RMSD was 4.2%, indicating good repeatability.

We then aimed at direct coupling of CEX and CIU to gain information on the conformation of chromatography-resolved protein populations. Similar to our previously described SEC–CIU optimization procedure,^22^ all critical CIU parameters were adapted to obtain successful hyphenation of CEX and CIU, including the acquisition time, magnitude, and number of CV steps. The CV in the trap was increased from 0 to 150 V in steps of 10 V. The maximum CV of 150 V was selected to minimize the risk of fragmentation and obtain high-quality data while still covering the most diagnostic CIU energy range of mAbs. Furthermore, a cone voltage of 80 V was sufficient for intact mAb analysis, whereas for the middle level, 60 V was chosen to prevent changes in mAb conformation (Figure S1). To find the optimal number of CV scans per run and the acquisition time, the CEX peak width (around 0.80 min) as well as the spectral quality was considered. We compared the effect of four CVs per chromatographic peak (each CV was applied for 20 s during which five scans of 4 s were recorded) with six CVs (15 s for each CV during which five scans of 3 s were recorded) (Figure S2). Even though the latter condition reduced the total analysis time (three 20 min CEX–CIU runs instead of four), noisy CIU features are obtained above 100 V due to a lower signal-to-noise ratio. Therefore, a total of four CEX–CIU runs (0–30, 40–70, 80–110, and 120–150 V) were chosen to record a complete high-quality CIU fingerprint (Figure 2). Importantly, the lowest voltages of the run covering the 0–30 V range should be recorded at the peak maximum due to low desolvation and trapping efficiencies at these low voltages together with a low-intensity signal at the beginning of the chromatographic peak (Figure S3). Compared to the previously reported SEC–CIU method,^22^ CEX–CIU required a longer analysis time to record the whole dataset from 0 to 150 V. More precisely, a complete CEX–CIU fingerprint will take 80 min (four times 20 min) compared to 15 min (three times 5 min) for SEC–CIU. However, CEX–CIU supplemented the CIU workflow with a chromatographic separation dimension rather than solely automated sample introduction in the MS instrument as for SEC–CIU, counterbalancing the longer acquisition times with improved separation power.

To benchmark our method development, we first applied the optimized CEX–CIU method to the analysis of the reference mAb trastuzumab, resulting in four different states within the whole CIU fingerprint (Figure 2b). The conformational transitions between these four different states were observed at 18.2 ± 0.2, 34 ± 2, and 91 ± 3 V, respectively. One feature of this fingerprint is the coexistence of unfolding states 2 and 3 over a wide voltage range (between 40 and 120 V). This particular CIU signature has already been reported for IgG1 fingerprints of high charge states (27+),^22^ being in good agreement with the isotype subclass of trastuzumab. The CIU fingerprints of the CEX-separated Fc and F(ab′)_2_ domains were recorded using similar experimental parameters (Figure S4). The Fc subunit (14+) showed two transitions at 27 ± 2 and 116 ± 3 V (Figure S4a). To provide more insights into the latter transition, a manual inspection of the mass spectra of the Fc subunit along with the analysis of the corresponding ATD profile was performed (Figure S5). According to these results, the upper limit of the Fc fingerprint was set to 130 V as further activation led to fragmentation of molecular ions and thus the total loss of the MS signal. As depicted in Figure S5, the most unfolded state (around 12 ms) starts to be populated at 30 V and becomes the most intense conformation in the ATD at 40 V. Beyond 90 V, this conformational state starts to be less populated, favoring the increase of an additional unfolding state with a shorter drift time (around 10 ms). Although further analysis should be carried out to confirm the structural mechanism behind this conformational change, these results could suggest that the Fc subunit of mAbs can undergo compaction under specific experimental conditions. For the F(ab′)_2_ part (21+), three transitions were observed giving rise to four progressively unfolding states (Figure S4b). These transitions are observed at 22.5 ± 0.1, 72 ± 7, and 128 ± 7 V, respectively.

Similar CIU fingerprints of mAbs and mAb subunits have been previously reported,^3,17,22^ indicating that the obtained CIU fingerprints of trastuzumab were not affected by the upfront CEX separation. The RMSD value between CEX–ESI–CIU and ESI–CIU at the intact level was 6.6% (Figure S6a), in line with differences observed in previous studies using the combination of SEC with CIU.^22^ The RMSD between the middle-level analyses using CEX–ESI–CIU and ESI–CIU was 6.5% (Figure S6b). In both cases, the obtained RMSD was similar to the RMSD values between replicates (Figure S6). The slight differences observed at the intact level between the inter- and intra-RMSD values can stem from the fact that both analyses were not recorded at the same flow rate (4 μL/min for ESI–CIU and 250 μL/min for CEX–ESI–CIU) along with the variation of the IM–MS signal transmission as a consequence of the chromatography peak elution when recording CIU fingerprints in combination with CEX. However, the RMSD values pinpoint that online CEX–CIU affords comparable and consistent CIU results, leading to the conclusion that neither the fractionation of the CIU recording nor the interaction with the CEX stationary phase significantly impairs the quality of the CIU fingerprints of therapeutic mAbs.

### Proof of Concept: CEX–CIU for Nearly Isobaric mAb Mixture Analysis

**Figure 3:**
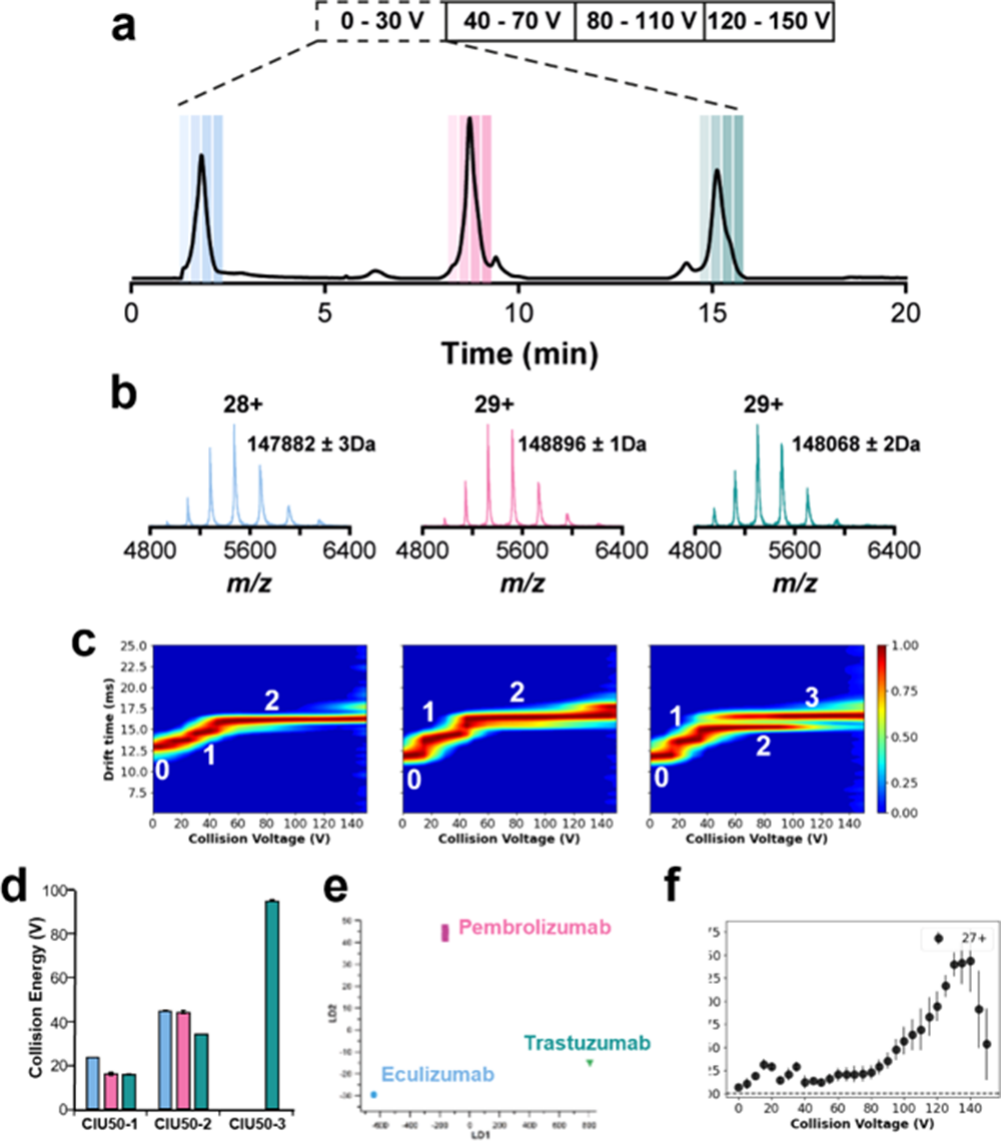
CEX–CIU for multiplex analysis of different mAbs, including eculizumab (IgG2/4), pembrolizumab (IgG4), and trastuzumab (IgG1). (a) CEX–UV chromatogram of a mixture showing the selected peaks for CIU analysis. During elution of the selected species, the CV is increased in four steps, where each CV is maintained for 20 s. (b) Mass spectra of the selected CEX peaks, including the experimental mass of the most abundant species. (c) CEX–CIU fingerprints of the mAbs of the 27+ charge state. CEX–CIU fingerprints of the 26+ and 28+ charge states can be found in Figures S7 and S8, respectively. (d) CIU50 values of the different detected transitions within the CIU fingerprints. (e) Linear determinant analysis of the three mAb CIU fingerprints. (f) UFS plot highlighting the critical CVs for mAb subclass identification.

Since the CEX–CIU approach was shown to be well suited to monitor the unfolding mechanism of different chromatography-selected populations, this analytical strategy was applied to a mixture of three therapeutic mAbs at the intact level, as a proof of concept of the methodology. The proposed application can be particularly interesting for biopharmaceutical companies since the simultaneous characterization of individual components contained in an mAb mixture is gaining more attention due to novel promising co-formulated mAb products.^29^ The selected mAbs belonged to different isotype subclasses exhibiting differences in inter-chain disulfide patterns. Previous studies have shown that the CIU mechanism strongly depends on the inter-chain disulfide pattern of mAbs, and therefore, the CIU fingerprints of these mAbs should show different unfolding energies and number of transitions. Three therapeutic mAbs with relatively similar molecular masses but substantially different pIs (i.e., 6.1 for eculizumab, 7.6 for pembrolizumab, and 9.1 for trastuzumab^30^) were chosen for the mAb mixture analysis. Thereby, the relatively small difference in molecular masses hampered studying unfolding patterns with classical direct infusion techniques or applying non-denaturing size-based separations, such as SEC.^22^ In this case, CEX–CIU is a suitable alternative, since mAbs can be separated based on pIs prior to the nMS analysis (Table S2). Additionally, this approach allows simultaneous monitoring of minor charge variants within the mAb mixture. The CEX gradient started by maintaining 0% B for 1 min to ensure elution of eculizumab, whereafter a linear increase from 30 to 70% B in 9 min enabled separation of pembrolizumab and trastuzumab. The obtained CEX chromatogram showed three (major) distinct peaks corresponding to the different mAbs (Figure 3a,b), where eculizumab elutes at 1.8 min, pembrolizumab at 8.9 min, and trastuzumab at 15.1 min.

After optimizing the CEX separation, the CIU fingerprints of the separated mAbs were recorded and compared (Figure 3c). Using this approach, four CEX–CIU runs of 20 min (in total, 80 min) were required to obtain complete CEX–CIU fingerprints for the three mAbs. A clear advantage of this approach is the low risk of interferences in the CIU pattern coming from different species since the three mAbs elute at different retention times. To illustrate the detected differences, the CIU50 values were calculated (Figure 3d). In the case of trastuzumab, three different unfolding events were observed, while only two transitions were detected for eculizumab and pembrolizumab. Thereby, a CIU50 value of 95.1 ± 0.2 V was determined for this last transition (CIU50-3), being the sole mAb that undergoes unfolding transition at collision energies higher than 50 V. The RMSD between trastuzumab measured as part of the mAb mixture and measured as single mAb was 6.1%, whereas the RSMDs between the different mAb subclasses within the mixture were ranging from 11.2 to 18.8% (Figure S9). To strengthen these results, linear discriminant analysis was performed (Figure 3e), showing the ability to distinguish the three mAbs based on their CIU features. This indicates that the CIU patterns of the mAbs are indeed different, and no additional variation was introduced upon mixing the mAbs. Additionally, the UFS plot pinpointed the 120–140 V region as the most diagnostic energy range to perform the inter-subclass comparison (Figure 3f), being in agreement with previous reports.^17,31^ After the analysis of these results, the benefit of the CEX–ESI–CIU approach is clearly shown to study a co-formulation mixture of therapeutic mAbs providing a separation of the different components in the first dimension (CEX) and subsequently recording the unfolding pattern of each protein population. The outcome of this approach not only improves the characterization of co-formulated mAbs without jeopardizing the chromatographic resolution but also reduces the overall analysis time by acquiring multiple CIU fingerprints at once.

### CEX–ESI–CIU To Monitor Differences in Gas-Phase Unfolding of mAb Glyco-Variants

As previous reports have shown that glycosylation impacts the gas-phase unfolding of mAbs,^18,32^ we applied the novel CEX–CIU platform to study the influence of glycosylation, particularly sialyation, on the gas-phase unfolding pattern as a particular type of charge variants.

**Figure 4:**
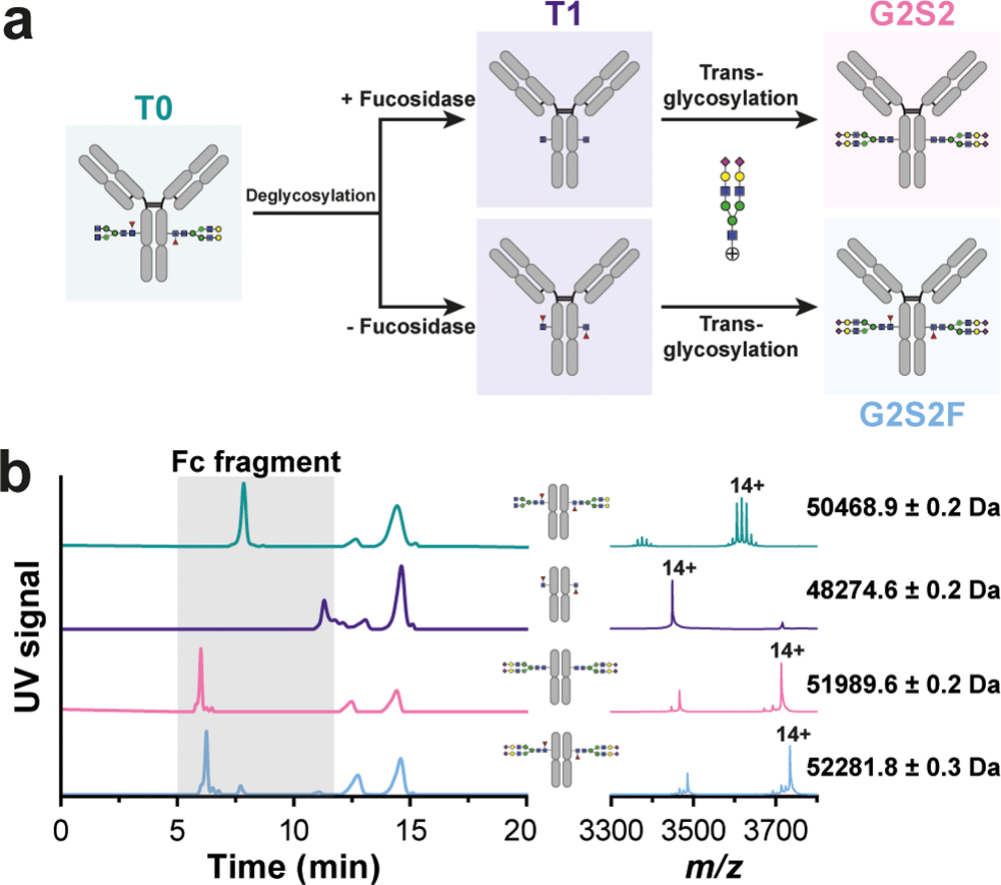
Modification of the glycan moiety of trastuzumab. (a) Schematic overview of the glycan remodeling workflow. The deglycosylation was performed with or without the presence of a fucosidase, resulting in different starting points for the transglycosylation (either GlcNAcFuc or GlcNAc). (b) CEX–UV separation of glycoengineered trastuzumab at the middle level highlighting the retention time shift of the Fc domains. Corresponding mass spectra of the different Fc subunits including the (major) experimental mass are presented. Mass spectra of the F(ab′)_2_ domain can be found in Figure S10.

The different glycoforms of trastuzumab were created using glycan-based enzymatic remodeling (see the Experimental Section for details on the procedure). The heterogeneous pool of glycans present on the initial product (T0) was first partially removed (EndoS2 treatment), leading to the deglycosylated mAb (T1) followed by replacement with a particular glycoform (G2S2 with or without core F) (Figure 4a). The remodeled glycans contained additional negative charges from the sialic acids, which resulted in modification of the protein surface charges. Since we were interested in differences in glycosylation located on the Fc part, the glycoengineered trastuzumab formats were digested with the IdeS enzyme, and Fc and F(ab′)_2_ subunits were further analyzed by CEX–CIU. As expected, the Fc fragments with remodeled glycans showed a shift to lower retention times in CEX, specifically 6.0 min for G2S2 and 6.3 min for G2S2F compared to 8.0 min for T0 (Figure 4b). Notably, the Fc fragment carrying G2S2F showed a minor peak around 7.8 min, indicating that the reaction was not complete and a minor amount of the T0 product was still present. However, due to the CEX baseline separation of the different Fc fragments, there was no inference of these species in the region where the CIU fingerprints were recorded. The peaks of the F(ab′)_2_ fragment eluted at the same retention time (14.5 min) for all samples and showed similar mass spectra, suggesting that no other modifications are induced on this part during the sample treatment as expected (Figure S10).

**Figure 5:**
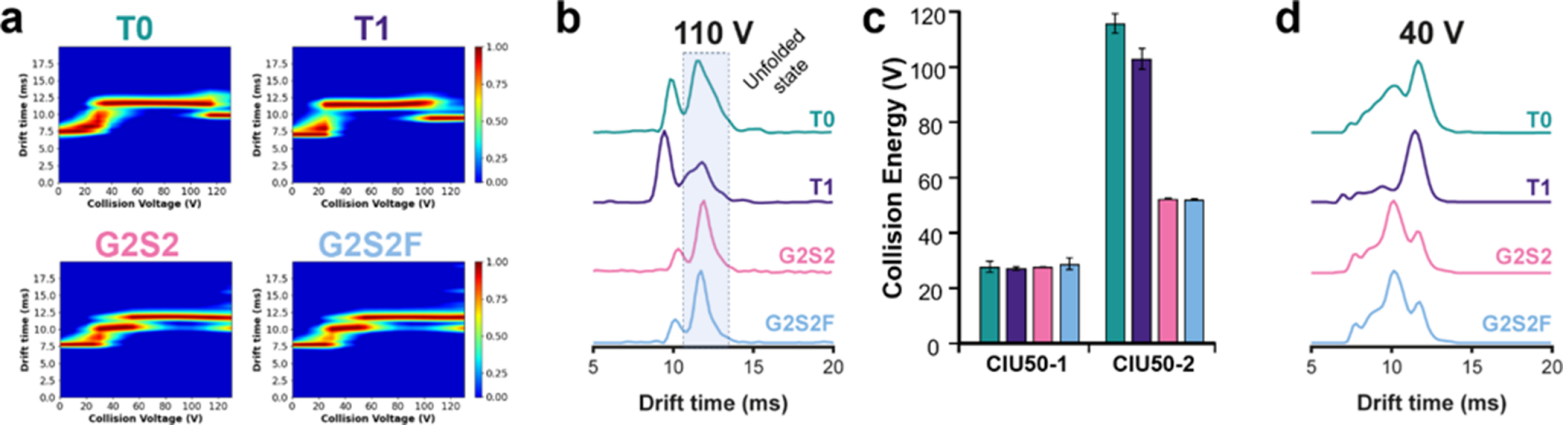
CEX–CIU experiments of middle-level trastuzumab samples subjected to glycan remodeling. (a) CIU fingerprints of the 14+ charge state of the Fc fragments carrying different glycans, including the initial (T0, green), deglycosylated (T1, purple), and end (G2S2, pink and G2S2F, blue) products. Differential plots including RMSD values can be found in Figure S11. (b) ATDs extracted at 110 V evidencing the higher relative intensity of the most unfolded conformation in the cases of G2S2 and G2S2F showing greater resistance to unfolding events. (c) CIU50 analysis of the samples revealing an intermediate transition only observed in the case of sialylated variants. (d) ATDs extracted at 40 V, showing that differences in the ATD profile can also be detected in the low-energy range of the CIU fingerprints. The F(ab′)_2_ CIU fingerprints showed no differences in resistance to gas-phase unfolding (Figure S12).

The CIU fingerprints of the Fc and F(ab′)_2_ fragments were acquired (Figures 5, S11, and S12). As previously mentioned, the Fc CIU fingerprints were recorded until 130 V to reduce ion activation and ensure enough IM signal intensity. However, substantial differences in unfolding behavior between glycoforms were observed in the fingerprints of the Fc domain in the 0–130 V range. This clearly highlights the benefit of CIU workflows over conventional IM–MS, which was not able to distinguish the different glycoforms in their ground state (Figure S13).

Comparing the CIU fingerprints of the glycosylated T0 product and the deglycosylated T1 product revealed the same number of transitions. Nevertheless, some differences can be observed regarding the gas-phase kinetic stability of the unfolding features of both subunits. For instance, the most unfolded conformation of T0 (12 ms) is the most populated conformation at 110 V, whereas for T1, this conformation undergoes transition at lower collision energies, and thus, the conformation observed at 10 ms is the most populated state at 110 V (Figure 5b,c). This indicates that deglycosylation of trastuzumab results in an Fc domain more prone to gas-phase unfolding, which is in agreement with previously performed differential scanning calorimetry (DSC) as well as CIU experiments.^18,20,33,34^ Regarding the CIU fingerprints of sialylated Fc variants, differential features can also be detected upon inspection of the unfolding patterns. Interestingly, an intermediate unfolding transition can be observed around 52 V for the G2S2 and G2S2F products, which was not observed for T0 or T1 (Figure 5a,c). Furthermore, the most unfolded conformation of those products (state 3) was still detected as the most intense conformation at the highest voltage (130 V). These results clearly pinpoint that the presence of sialic acid glycans confers higher unfolding resistance to the Fc domain of the mAb. According to our results, the presence of core fucoses on these glycans does not influence the gas-phase unfolding mechanism of these products reflected in very similar unfolding patterns. The latter was also confirmed by previously reported DSC data, where afucosylated IgG1 glycoforms did not show different thermal unfolding compared to the fucosylated counterpart.^35,36^ Overall, the differences between the glycan-remodeled Fc subunits were highlighted when performing differential analysis between the fingerprints (Figure S11). Upon pairwise comparison, RMSD values greater than 14% were obtained (excluding the comparison between G2S2 and G2S2F, where almost no differences were observed), while the variation of the technical replicates was lower than 6%. According to the UFS plot, the most diagnostic voltages to differentiate the glycoengineered Fc domains were between 100 and 120 V (Figures S11c and 5b). In addition, the Fc CIU features showed differences in the 20–60 V range. The extracted ATDs at 40 V (Figure 5d) revealed clear-cut differences between the glycoengineered Fc domains compared to T0 and T1, with a different number of coexisting populations along with different relative intensities, allowing the categorization of the Fc species. As above mentioned, the presence of fucose does not significantly alter the Fc gas-phase unfolding mechanism and hence the high similarity of G2S2 and G2S2F ATD profiles.

The CIU patterns of the F(ab′)_2_ subunits revealed the same number of unfolding transitions along with very similar CIU50 values. Upon pairwise comparison of the F(ab′)_2_ fingerprints, the RMSD values were close to those obtained upon technical replicates which evidenced the similarities between the CIU fingerprints of those domains, suggesting that the F(ab′)_2_ unfolding mechanism was not affected by the Fc glycan scaffold (Figure S12). The same conclusion was drawn in previous studies based on DSC measurements, demonstrating that the melting temperatures of the Fab and CH3 domains of different N-glycosylated mAbs were not affected by the presence of different glycan moieties.^37^ Altogether, the CEX–CIU approach was perfectly adapted to monitor structural modifications of mAbs while simultaneously obtaining information on the unfolding mechanism of the chromatography-selected species. The proposed experimental setup allowed the correlation between the glycan-engineered scaffold and unfolding signatures in the CIU fingerprints, providing valuable information on the influence of local structure alteration (i.e., altered glycan moieties) on the unfolding pattern of the Fc subunit of trastuzumab.

## Conclusions

In this study, we aimed at the innovative hyphenation of CEX with CIU experiments to provide thorough characterization of therapeutic mAbs. This experimental setup combined the benefits of CEX separation to afford information about protein heterogeneity together with the CIU analysis to allow the characterization of those protein populations beyond their folded state. After developing this approach at both the intact and middle levels, we showed that the analytical strategy is well suited to record the global conformation and the CIU unfolding pattern of different CEX-separated species without affecting the resolution and chromatography separation capabilities. Moreover, the recorded CEX–CIU fingerprints contained a similar level of information compared to those obtained under classical ESI–CIU conditions.

To show the applicability of our innovative CEX–CIU platform, we focused on a mixture of three therapeutic mAbs close in mass but with different pIs and representatives from different subclasses (with different inter-chain disulfide patterns). The three CIU fingerprints exhibited different unfolding transitions leading to the conclusion that CEX–CIU was able to maintain the key features of the unfolding mechanism of each mAb, thus allowing the differentiation of those proteins. Furthermore, these results showed that the overall CEX–CIU analysis time could be significantly reduced when dealing with therapeutic mAb mixtures. Additionally, CEX–CIU coupling emerged as an appealing alternative in the particular case of isobaric mAb mixtures since these proteins cannot be separated using SEC–CIU. In this case, CEX–CIU affords a baseline-resolved separation according to mAb pIs; hence, CEX–CIU combines the synergy of separation based on pIs and CIU recording.

Besides, applicability of CEX–CIU to obtain glycan-variant-specific information was investigated by studying the gas-phase unfolding of Fc regions carrying different glycan moieties. The Fc CIU patterns showed variations in gas-phase conformational transitions as a result of altered glycosylation, while conventional IM–MS measurements were unable to provide clear conclusions (Figure S13), highlighting the suitability of the CIU approach to study protein populations with similar CCS values. Based on the CIU patterns, it was shown that deglycosylated Fc fragments were more prone to unfolding events, while remodeled glycoforms showed increased resistance to gas-phase unfolding.

Altogether, the results provided in this study evidence that hyphenation of CEX with CIU has the potential to provide in-depth characterization of therapeutic mAbs. First, CEX–CIU affords practical benefits, such as automation of CIU fingerprint recording while maintaining pI separation without any prior sample preparation, leading to an increased throughput. More importantly, CEX–CIU not only enables the identification of protein populations using classical CEX–nMS conditions but also offers the possibility of simultaneous conformational characterization of those chromatography-resolved populations. In future, the application of this approach could be further expanded to low-abundance charge variant analysis, including deamidation and oxidation, either in drug products or in forced degraded samples. Both the level of structural information and the streamlined analysis afforded by the CEX–CIU approach make this technique particularly interesting to be integrated into R&D laboratories performing analysis of mAb-derived proteins.

## Supporting Information Available

Influence of cone voltages on middle-level analysis; CEX–CIU fingerprints with four or six IM–MS functions; effect of the order of applied CVs during peak elution; CEX–CIU of middle-level trastuzumab; individual mass spectra and ATDs at different energies of the Fc subunit; comparison of ESI–CIU and CEX–ESI–CIU experiments; CIU50 analysis of multiplexed mAbs (27+); CIU fingerprints and CIU50 analysis of multiplexed mAbs (26+); CIU fingerprints and CIU50 analysis of multiplexed mAbs (28+); mass spectra of F(ab′)_2_ fragments before and after glycan remodeling; RMSD plots of Fc fragments with remodeled glycosylation; CEX–CIU fingerprints of F(ab′)_2_ domains before and after glycan remodeling; IM–nMS analysis of Fc domains with differences in glycosylation; retention times and relative peak areas of CEX separation; and characteristics of multiplexed mAbs (PDF)

## References

[ref1] Carter P. J.; Lazar G. A. Next generation antibody drugs: pursuit of the ‘high-hanging fruit’. Nat. Rev. Drug Discovery 2018, 17, 197–223. 10.1038/nrd.2017.227. 29192287

[ref2] Kaplon H.; Chenoweth A.; Crescioli S.; Reichert J. M. Antibodies to watch in 2022. mAbs 2022, 14, 2014296 10.1080/19420862.2021.2014296. 35030985 PMC8765076

[ref3] Hernandez-Alba O.; Wagner-Rousset E.; Beck A.; Cianferani S. Native Mass Spectrometry, Ion Mobility, and Collision-Induced Unfolding for Conformational Characterization of IgG4 Monoclonal Antibodies. Anal. Chem. 2018, 90, 8865–8872. 10.1021/acs.analchem.8b00912. 29956914

[ref4] Beyer B.; Schuster M.; Jungbauer A.; Lingg N. Microheterogeneity of Recombinant Antibodies: Analytics and Functional Impact. Biotechnol. J. 2018, 13, 1700476 10.1002/biot.201700476. 28862393

[ref5] Füssl F.; Cook K.; Scheffler K.; Farrell A.; Mittermayr S.; Bones J. Charge Variant Analysis of Monoclonal Antibodies Using Direct Coupled pH Gradient Cation Exchange Chromatography to High-Resolution Native Mass Spectrometry. Anal. Chem. 2018, 90, 4669–4676. 10.1021/acs.analchem.7b05241. 29494133

[ref6] Liu H.; Ponniah G.; Zhang H. M.; Nowak C.; Neill A.; Gonzalez-Lopez N.; Patel R.; Cheng G.; Kita A. Z.; Andrien B. In vitro and in vivo modifications of recombinant and human IgG antibodies. mAbs 2014, 6, 1145–1154. 10.4161/mabs.29883. 25517300 PMC4622420

[ref7] Haberger M.; Heidenreich A. K.; Hook M.; Fichtl J.; Lang R.; Cymer F.; Adibzadeh M.; Kuhne F.; Wegele H.; Reusch D.; Bonnington L.; Bulau P. Multiattribute Monitoring of Antibody Charge Variants by Cation-Exchange Chromatography Coupled to Native Mass Spectrometry. J. Am. Soc. Mass Spectrom. 2021, 32, 2062–2071. 10.1021/jasms.0c00446. 33687195

[ref8] Fekete S.; Beck A.; Veuthey J. L.; Guillarme D. Ion-exchange chromatography for the characterization of biopharmaceuticals. J. Pharm. Biomed. Anal. 2015, 113, 43–55. 10.1016/j.jpba.2015.02.037. 25800161

[ref9] van Schaick G.; Haselberg R.; Somsen G. W.; Wuhrer M.; Domínguez-Vega E. Studying protein structure and function by native separation–mass spectrometry. Nat. Rev. Chem. 2022, 6, 215–231. 10.1038/s41570-021-00353-7. 37117432

[ref10] Yan Y.; Liu A. P.; Wang S.; Daly T. J.; Li N. Ultrasensitive Characterization of Charge Heterogeneity of Therapeutic Monoclonal Antibodies Using Strong Cation Exchange Chromatography Coupled to Native Mass Spectrometry. Anal. Chem. 2018, 90, 13013–13020. 10.1021/acs.analchem.8b03773. 30280893

[ref11] Murisier A.; Duivelshof B. L.; Fekete S.; Bourquin J.; Schmudlach A.; Lauber M. A.; Nguyen J. M.; Beck A.; Guillarme D.; D’Atri V. Towards a simple on-line coupling of ion exchange chromatography and native mass spectrometry for the detailed characterization of monoclonal antibodies. J. Chromatogr. A 2021, 1655, 462499 10.1016/j.chroma.2021.462499. 34487883

[ref12] Zhang L.; Patapoff T.; Farnan D.; Zhang B. Improving pH gradient cation-exchange chromatography of monoclonal antibodies by controlling ionic strength. J. Chromatogr. A 2013, 1272, 56–64. 10.1016/j.chroma.2012.11.060. 23253120

[ref13] Bailey A. O.; Han G.; Phung W.; Gazis P.; Sutton J.; Josephs J. L.; Sandoval W. Charge variant native mass spectrometry benefits mass precision and dynamic range of monoclonal antibody intact mass analysis. mAbs 2018, 10, 1214–1225. 10.1080/19420862.2018.1521131. 30339478 PMC6284562

[ref14] Di Marco F.; Berger T.; Esser-Skala W.; Rapp E.; Regl C.; Huber C. G. Simultaneous Monitoring of Monoclonal Antibody Variants by Strong Cation-Exchange Chromatography Hyphenated to Mass Spectrometry to Assess Quality Attributes of Rituximab-Based Biotherapeutics. Int. J. Mol. Sci. 2021, 22, 9072 10.3390/ijms22169072. 34445776 PMC8396523

[ref15] Dixit S. M.; Polasky D. A.; Ruotolo B. T. Collision induced unfolding of isolated proteins in the gas phase: past, present, and future. Curr. Opin. Chem. Biol. 2018, 42, 93–100. 10.1016/j.cbpa.2017.11.010. 29207278 PMC5828980

[ref16] Pringle S. D.; Giles K.; Wildgoose J. L.; Williams J. P.; Slade S. E.; Thalassinos K.; Bateman R. H.; Bowers M. T.; Scrivens J. H. An investigation of the mobility separation of some peptide and protein ions using a new hybrid quadrupole/travelling wave IMS/oa-ToF instrument. Int. J. Mass Spectrom. 2007, 261, 1–12. 10.1016/j.ijms.2006.07.021.

[ref17] Botzanowski T.; Hernandez-Alba O.; Malissard M.; Wagner-Rousset E.; Desligniere E.; Colas O.; Haeuw J. F.; Beck A.; Cianferani S. Middle Level IM-MS and CIU Experiments for Improved Therapeutic Immunoglobulin Subclass Fingerprinting. Anal. Chem. 2020, 92, 8827–8835. 10.1021/acs.analchem.0c00293. 32453570

[ref18] Tian Y.; Han L.; Buckner A. C.; Ruotolo B. T. Collision Induced Unfolding of Intact Antibodies: Rapid Characterization of Disulfide Bonding Patterns, Glycosylation, and Structures. Anal. Chem. 2015, 87, 11509–11515. 10.1021/acs.analchem.5b03291. 26471104

[ref19] Ferguson C. N.; Gucinski-Ruth A. C. Evaluation of Ion Mobility-Mass Spectrometry for Comparative Analysis of Monoclonal Antibodies. J. Am. Soc. Mass Spectrom. 2016, 27, 822–833. 10.1007/s13361-016-1369-1. 26988372

[ref20] Deslignière E.; Ehkirch A.; Duivelshof B. L.; Toftevall H.; Sjogren J.; Guillarme D.; D’Atri V.; Beck A.; Hernandez-Alba O.; Cianferani S. State-of-the-Art Native Mass Spectrometry and Ion Mobility Methods to Monitor Homogeneous Site-Specific Antibody-Drug Conjugates Synthesis. Pharmaceuticals 2021, 14, 498 10.3390/ph14060498. 34073805 PMC8225019

[ref21] Botzanowski T.; Erb S.; Hernandez-Alba O.; Ehkirch A.; Colas O.; Wagner-Rousset E.; Rabuka D.; Beck A.; Drake P. M.; Cianferani S. Insights from native mass spectrometry approaches for top- and middle-level characterization of site-specific antibody-drug conjugates. mAbs 2017, 9, 801–811. 10.1080/19420862.2017.1316914. 28406343 PMC5524152

[ref22] Deslignière E.; Ehkirch A.; Botzanowski T.; Beck A.; Hernandez-Alba O.; Cianferani S. Toward Automation of Collision-Induced Unfolding Experiments through Online Size Exclusion Chromatography Coupled to Native Mass Spectrometry. Anal. Chem. 2020, 92, 12900–12908. 10.1021/acs.analchem.0c01426. 32886492

[ref23] Polasky D. A.; Dixit S. M.; Fantin S. M.; Ruotolo B. T. CIUSuite 2: Next-Generation Software for the Analysis of Gas-Phase Protein Unfolding Data. Anal. Chem. 2019, 91, 3147–3155. 10.1021/acs.analchem.8b05762. 30668913

[ref24] Migas L. G.; France A. P.; Bellina B.; Barran P. E. ORIGAMI: A software suite for activated ion mobility mass spectrometry (aIM-MS) applied to multimeric protein assemblies. Int. J. Mass Spectrom. 2018, 427, 20–28. 10.1016/j.ijms.2017.08.014.

[ref25] Bush M. F.; Hall Z.; Giles K.; Hoyes J.; Robinson C. V.; Ruotolo B. T. Collision Cross Sections of Proteins and Their Complexes: A Calibration Framework and Database for Gas-Phase Structural Biology. Anal. Chem. 2010, 82, 9557–9565. 10.1021/ac1022953. 20979392

[ref26] Harris R. J.; Kabakoff B.; Macchi F. D.; Shen F. J.; Kwong M.; Andya J. D.; Shire S. J.; Bjork N.; Totpal K.; Chen A. B. Identification of multiple sources of charge heterogeneity in a recombinant antibody. J. Chromatogr. B: Biomed. Sci. Appl. 2001, 752, 233–245. 10.1016/S0378-4347(00)00548-X. 11270864

[ref27] von Pawel-Rammingen U.; Johansson B. P.; Bjork L. IdeS, a novel streptococcal cysteine proteinase with unique specificity for immunoglobulin G. EMBO J. 2002, 21, 1607–1615. 10.1093/emboj/21.7.1607. 11927545 PMC125946

[ref28] Wagner-Rousset E.; Fekete S.; Morel-Chevillet L.; Colas O.; Corvaia N.; Cianferani S.; Guillarme D.; Beck A. Development of a fast workflow to screen the charge variants of therapeutic antibodies. J. Chromatogr. A 2017, 1498, 147–154. 10.1016/j.chroma.2017.02.065. 28400066

[ref29] Kim J.; Kim Y. J.; Cao M.; De Mel N.; Albarghouthi M.; Miller K.; Bee J. S.; Wang J.; Wang X. Analytical characterization of coformulated antibodies as combination therapy. mAbs 2020, 12, 1738691 10.1080/19420862.2020.1738691. 32138591 PMC7153825

[ref30] Goyon A.; Excoffier M.; Janin-Bussat M. C.; Bobaly B.; Fekete S.; Guillarme D.; Beck A. Determination of isoelectric points and relative charge variants of 23 therapeutic monoclonal antibodies. J. Chromatogr. B Anal. Technol. Biomed. Life Sci. 2017, 1065–1066, 119–128. 10.1016/j.jchromb.2017.09.033. 28961486

[ref31] Vallejo D. D.; Polasky D. A.; Kurulugama R. T.; Eschweiler J. D.; Fjeldsted J. C.; Ruotolo B. T. A Modified Drift Tube Ion Mobility-Mass Spectrometer for Charge-Multiplexed Collision-Induced Unfolding. Anal. Chem. 2019, 91, 8137–8146. 10.1021/acs.analchem.9b00427. 31194508

[ref32] Tian Y.; Ruotolo B. T. Collision induced unfolding detects subtle differences in intact antibody glycoforms and associated fragments. Int. J. Mass Spectrom. 2018, 425, 1–9. 10.1016/j.ijms.2017.12.005.

[ref33] Zheng K.; Bantog C.; Bayer R. The impact of glycosylation on monoclonal antibody conformation and stability. mAbs 2011, 3, 568–576. 10.4161/mabs.3.6.17922. 22123061 PMC3242843

[ref34] Upton R.; Migas L. G.; Pacholarz K. J.; Beniston R. G.; Estdale S.; Firth D.; Barran P. E. Hybrid mass spectrometry methods reveal lot-to-lot differences and delineate the effects of glycosylation on the tertiary structure of Herceptin(R). Chem. Sci. 2019, 10, 2811–2820. 10.1039/C8SC05029E. 30997002 PMC6425993

[ref35] Zheng K.; Yarmarkovich M.; Bantog C.; Bayer R.; Patapoff T. W. Influence of glycosylation pattern on the molecular properties of monoclonal antibodies. mAbs 2014, 6, 649–658. 10.4161/mabs.28588. 24662970 PMC4011909

[ref36] Houde D.; Peng Y.; Berkowitz S. A.; Engen J. R. Post-translational modifications differentially affect IgG1 conformation and receptor binding. Mol. Cell. Proteomics 2010, 9, 1716–1728. 10.1074/mcp.M900540-MCP200. 20103567 PMC2938052

[ref37] Wada R.; Matsui M.; Kawasaki N. Influence of N-glycosylation on effector functions and thermal stability of glycoengineered IgG1 monoclonal antibody with homogeneous glycoforms. mAbs 2019, 11, 350–372. 10.1080/19420862.2018.1551044. 30466347 PMC6380427

