# Supplementary material for: Online Collision-Induced Unfolding of Therapeutic Monoclonal Antibody Glyco-Variants through Direct Hyphenation of Cation Exchange Chromatography with Native Ion Mobility–Mass Spectrometry
Source: Anal Chem. 2023 Feb 15;95(8):3932–9. doi: 10.1021/acs.analchem.2c03163 (PMC9979139; doi:10.1021/acs.analchem.2c03163)
Supplement: Supplementary file 1 — ac2c03163_si_001.pdf [file ac2c03163_si_001.pdf]

Supporting Information

Online collision-induced unfolding of therapeutic monoclonal antibody glyco-variants through direct hyphenation of cation exchange chromatography with native IM-MS

Guusje van Schaick [1], Elena Domínguez-Vega [1], Jérôme Castel [2,3], Manfred Wuhrer [1], Oscar Hernandez-Alba [2,3], Sarah Cianferani [2,3*]

[1] Center for Proteomics and Metabolomics, Leiden University Medical Center, Albinusdreef 2, 2333 ZA, Leiden, the Netherlands
[2] Laboratoire de Spectrométrie de Masse BioOrganique, IPHC UMR 7178, Université de Strasbourg, CNRS, Strasbourg, France.
[3] Infrastructure Nationale de Protéomique ProFI, FR2048 CNRS CEA, Strasbourg, France.

* corresponding author

Table of contents **Page**

**Figure S1**	Influence of cone voltages on middle-level analysis	S2
**Figure S2**	CEX-CIU fingerprints with 4 or 6 IM-MS functions	S3
**Figure S3**	Effect of order of applied CVs during peak elution	S4
**Figure S4**	CEX-CIU of middle-level trastuzumab	S5
**Figure S5**	Individual mass spectra and ATDs at different energies of Fc subunit	S6
**Figure S6**	Comparison of ESI-CIU and CEX-ESI-CIU experiments	S7
**Figure S7**	CIU50 analysis and RMSD plots of multiplexed mAbs (27+)	S8
**Figure S8**	CIU50 analysis and RMSD plots of multiplexed mAbs (26+)	S9
**Figure S9**	CIU50 analysis and RMSD plots of multiplexed mAbs (28+)	S10
**Figure S10**	Mass spectra F(ab')$_2$ fragments before and after glycan remodeling	S11
**Figure S11**	RMSD plots Fc fragment after glycan remodeling	S12
**Figure S12**	CIU fingerprints of F(ab')$_2$ domains before and after glycan remodeling	S13
**Figure S13**	IM−nMS analysis of Fc domains with differences in glycosylation	S14
**Table S1**	Retention times and relative peak area of the CEX separation	S15
**Table S2**	Characteristics of the multiplexed mAbs	S16
**References**		S17

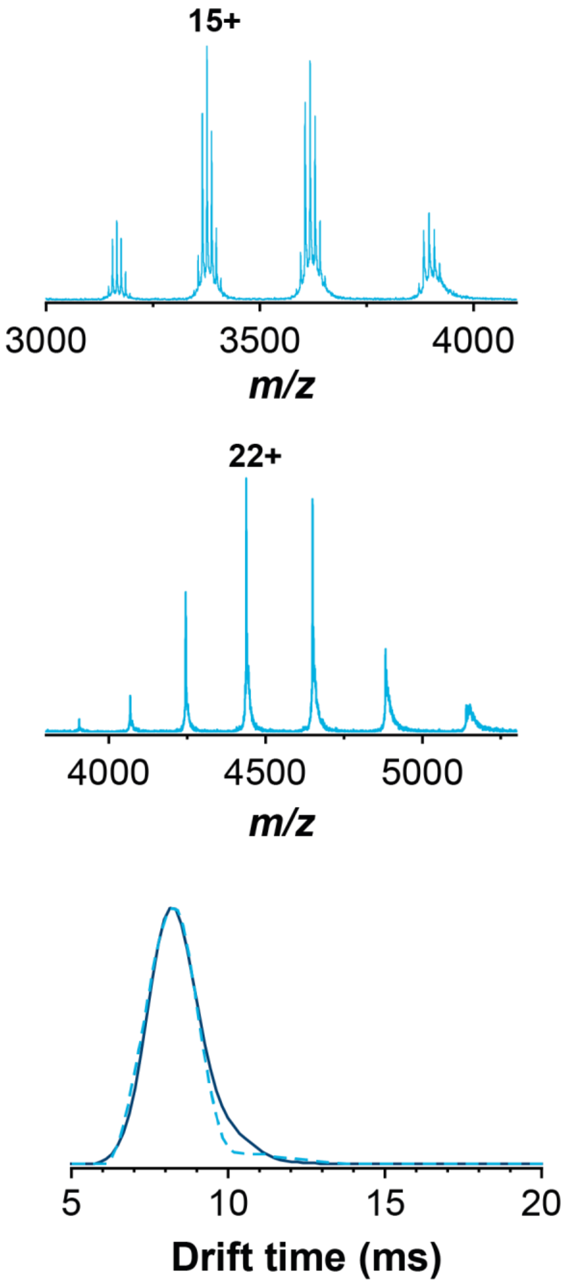

Figure S1. Mass spectra of Fc and F(ab’)$_2$ domain of trastuzumab after IdeS digestion and ATDs (collision voltage of 0V) of the Fc fragment measured with a cone voltage of 60 V (light blue) and with a cone voltage of 80 V (dark blue). The upper spectra correspond to Fc fragment and lower spectra to the F(ab’)$_2$ domain. The most intense charge state is indicated.

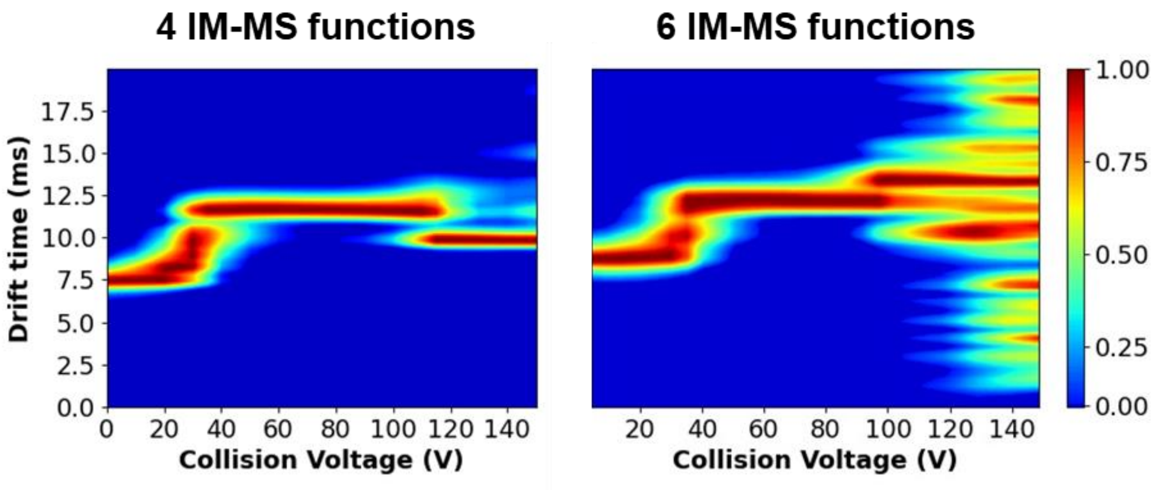

Figure S2. The CEX-CIU fingerprints of the Fc part of trastuzumab obtained using four or six IM-MS functions per CEX peak.

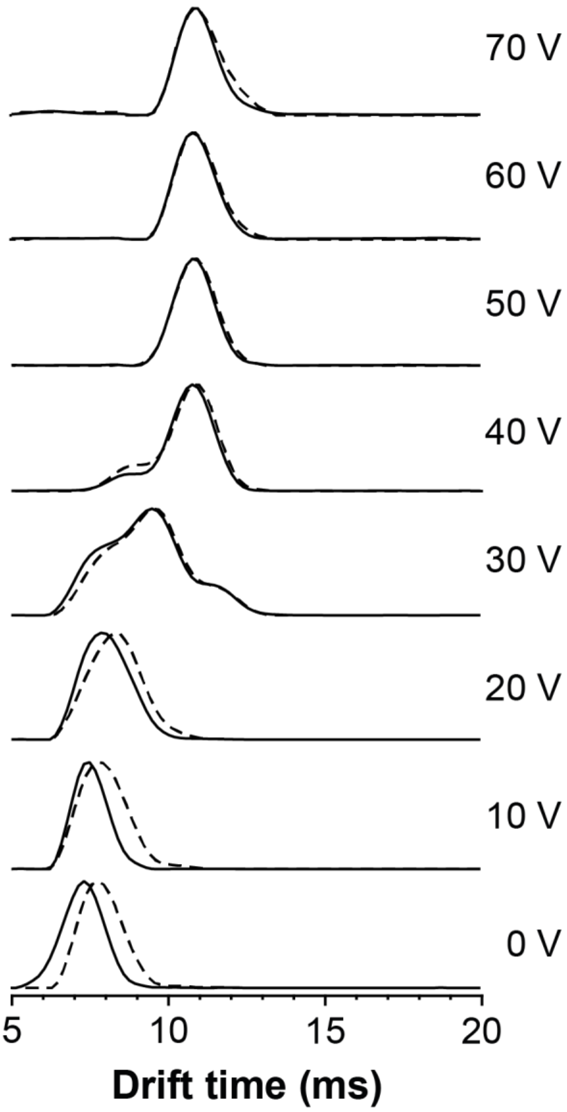

Figure S3. The ATDs of the Fc fragment of trastuzumab after IdeS digestion (14+ charge state) measured by increasing the CV stepwise during elution of the peak (solid line) and by recording the CVs in the following order 30-0-10-20 V and 70-40-50-60 (dotted line). As shown in the figure, ATDs drift times can vary when the energy values are applied randomly (dotted line) if the energy voltages are aligned at the beginning of the chromatographic peak due to a combination of low MS and IM-MS signal. This difference is not observed when higher energies are applied.

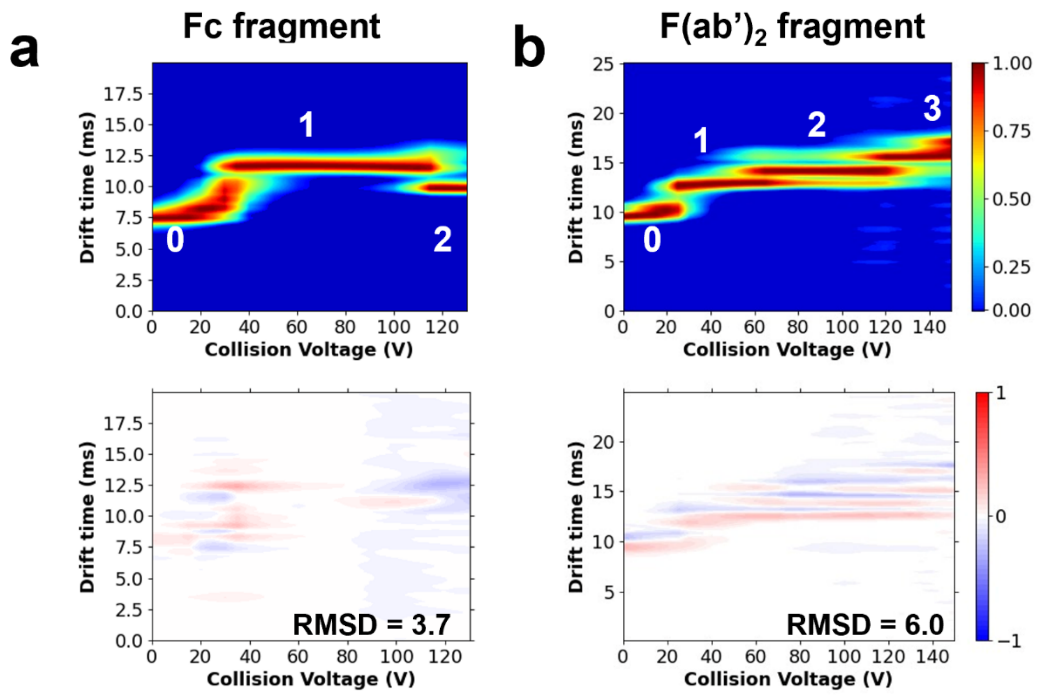

Figure S4. CEX-CIU fingerprints and replicate RMSD plot of the 14+ charge state of the Fc fragment (**a**) and the 21+ charge state of the F(ab')$_2$ fragment (**b**). The corresponding UV chromatogram can be found in **Figure 1b**. The detected conformational states are numbered.

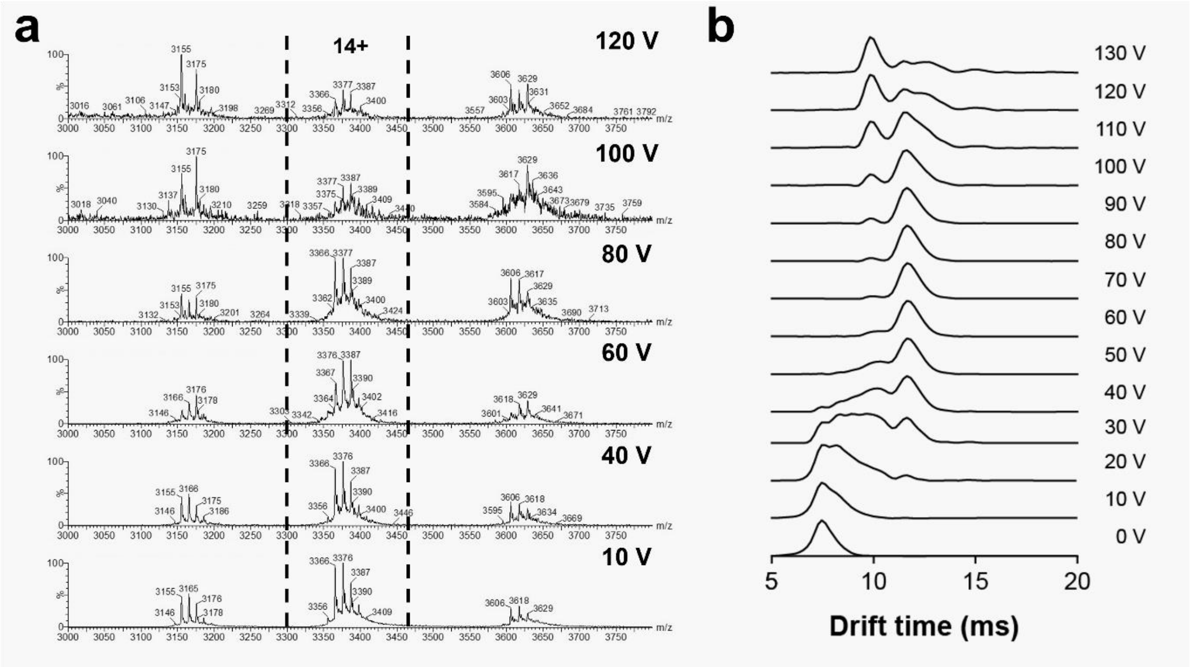

Figure S5. Mass spectra and ATDs distribution of the Fc subunit of trastuzumab at different collision energies. (**a**) The mass spectra show the intensities of the 15+, 14+, and 13+ charge states of the Fc subunit. The 14+ charge state was selected to record the CIU fingerprint. (**b**) Extracted arrival time distributions of the 14+ Fc at each individual collision energy (from 0 to 130V). The individual ATDs were used to build the fingerprint of the subunit.

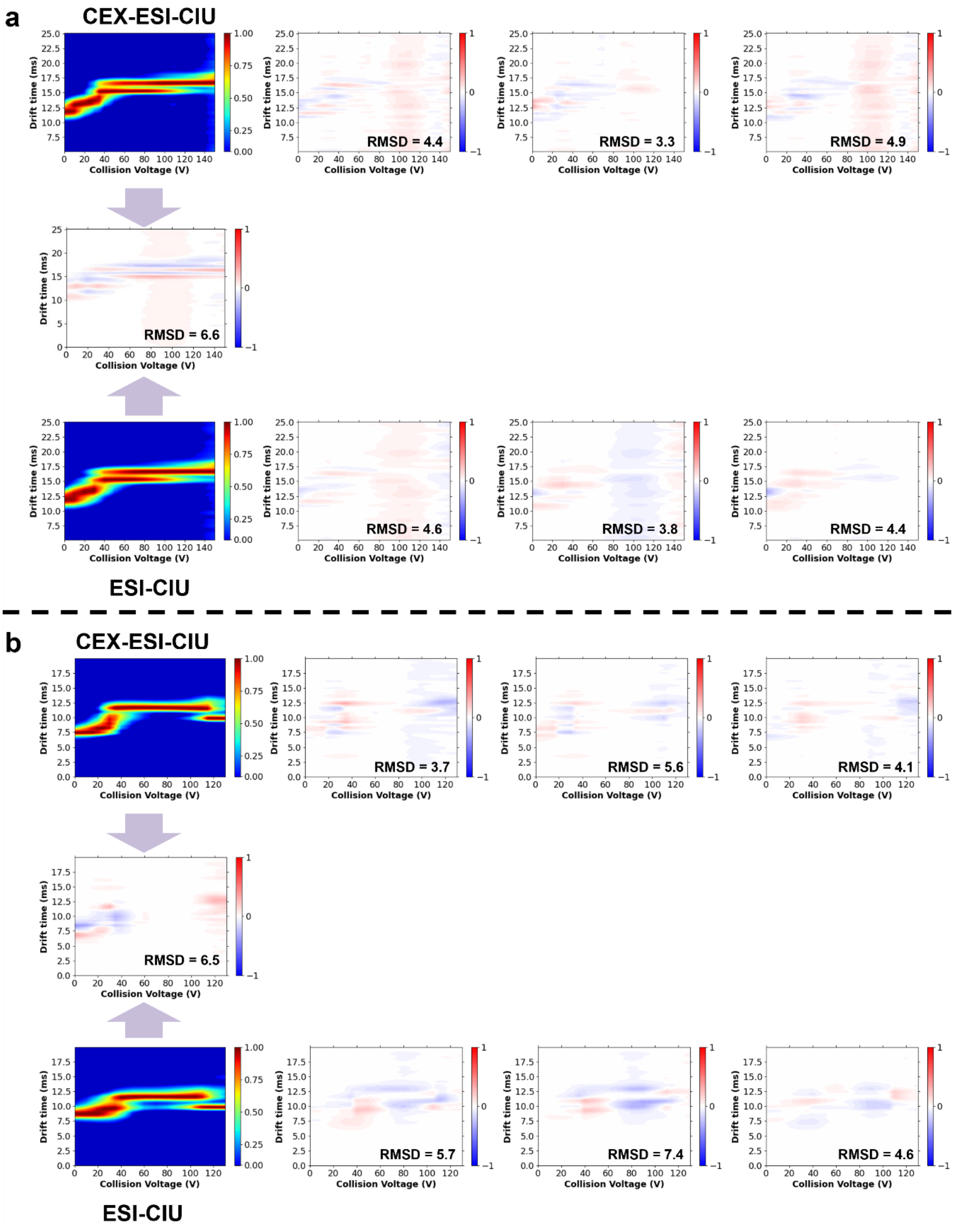

Figure S6. Comparison of CEX-ESI-CIU experiments with ESI-CIU data at the intact (**a**) and middle level (**b**). The differential plots between the CEX-ESI-CIU and ESI-CIU fingerprints show high similarities with RMSDs of 6.6 (intact) and 5.0 (Fc fragment). The replicate RMSD plots are provided for the presented CIU fingerprint, including the RMSD values. The intact CIU fingerprint is of the 27+ charge state and the Fc fragment CIU fingerprint is of the 14+ charge state.

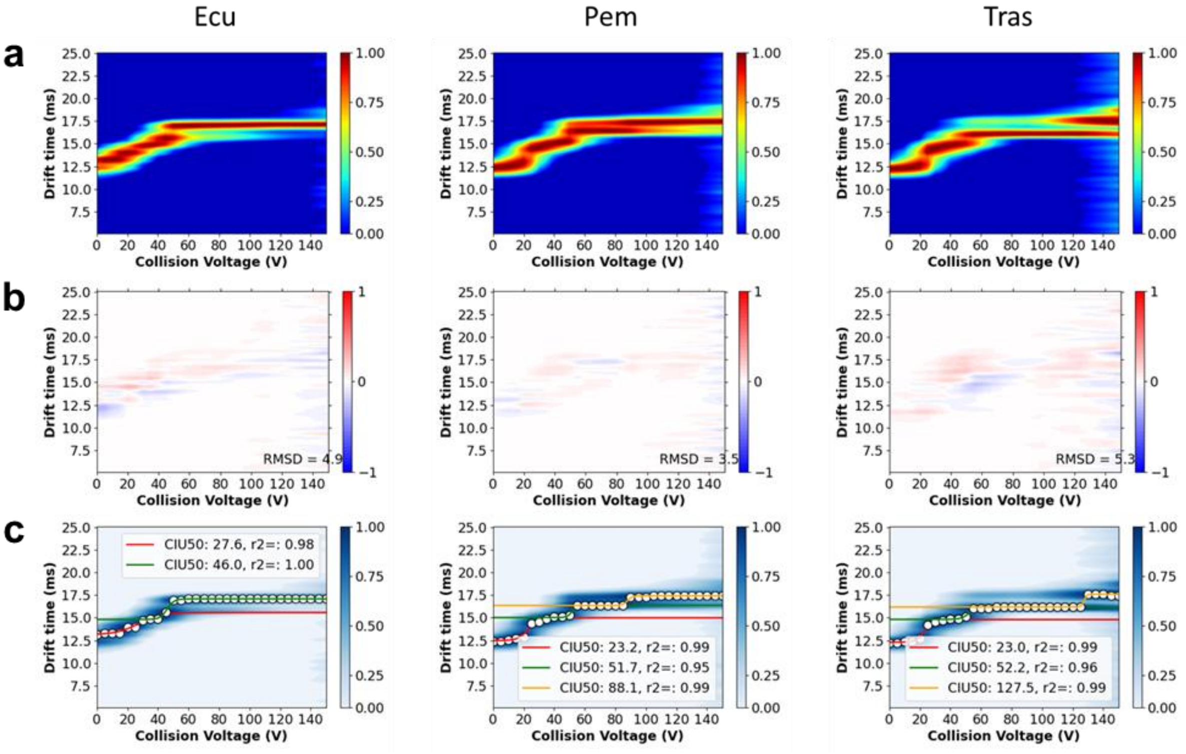

Figure S7. CIU experiments at the multiplexed mAbs (eculizumab, pembrolizumab, and trastuzumab) at the intact level for the 26+ charge state, including CIU fingerprints (**a**), replicate RMSD plots (**b**), and CIU50 analyses (**c**). The RMSD plots indicated the variation between the CIU fingerprints of the measured replicates.

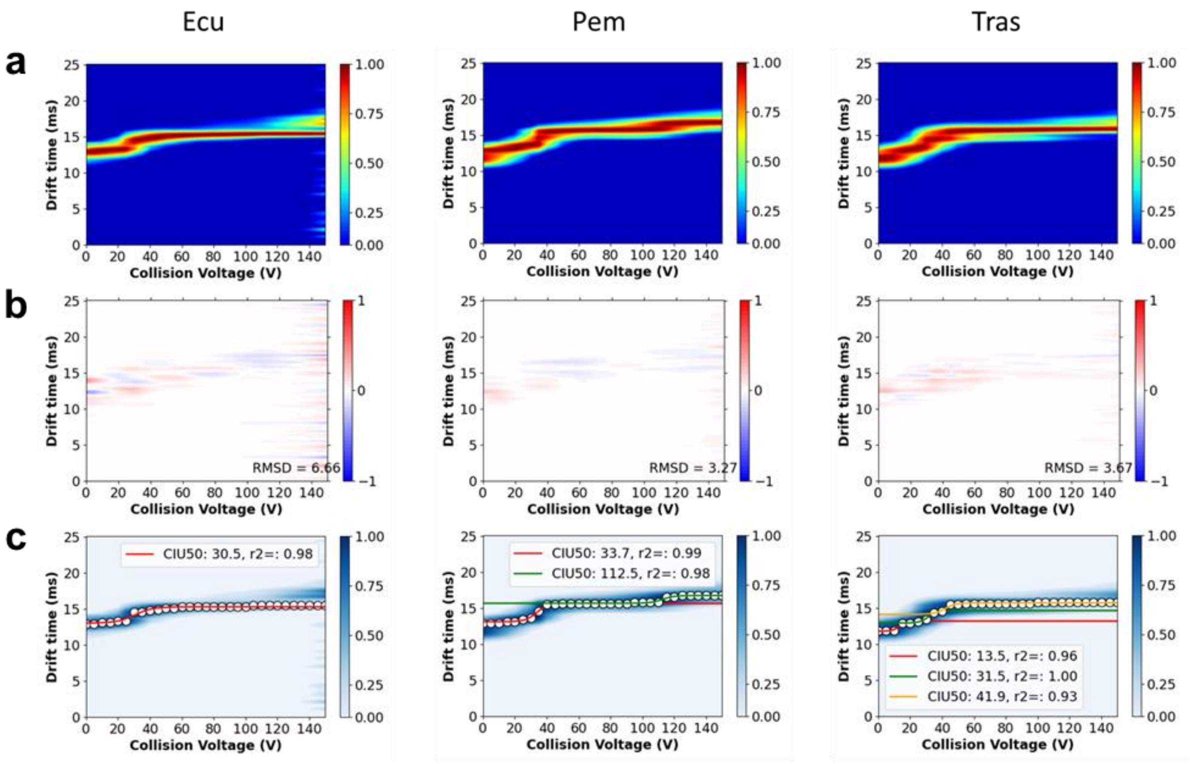

Figure S8. CIU experiments at the multiplexed mAbs (eculizumab, pembrolizumab, and trastuzumab) at the intact level for the 28+ charge state, including CIU fingerprints (**a**), replicate RMSD plots (**b**), and CIU50 analyses (**c**). The RMSD plots indicated the variation between the CIU fingerprints of the measured replicates.

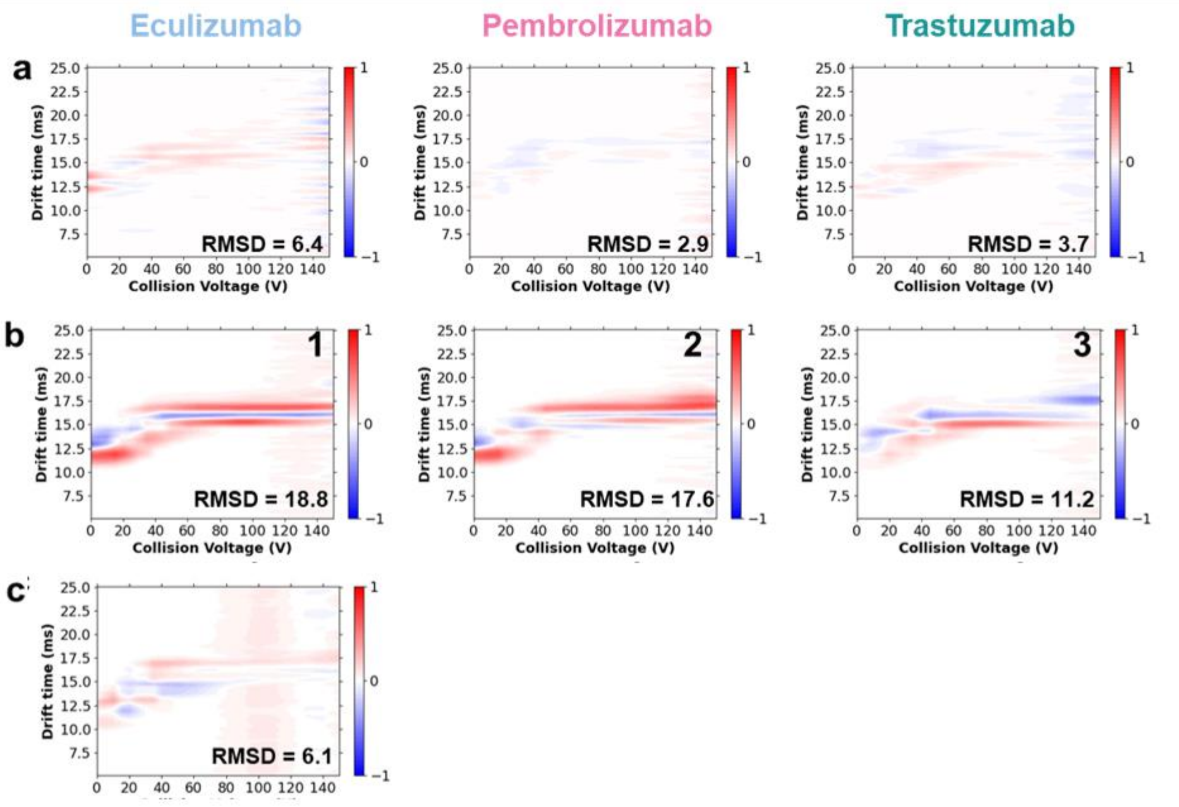

Figure S9. CIU experiments of the multiplexed mAbs at the intact level for the 27+ charge state. (**a**) Replicate RMSD plots of the measured mAbs in the mixture. (**b**) RMSD plots between the different mAbs, where 1 = eculizumab and trastuzumab, 2 = eculizumab and pembrolizumab, and 3 = pembrolizumab and trastuzumab. (**c**) Comparison of CIU fingerprint of trastuzumab recorded as a single mAb and in a mixture.

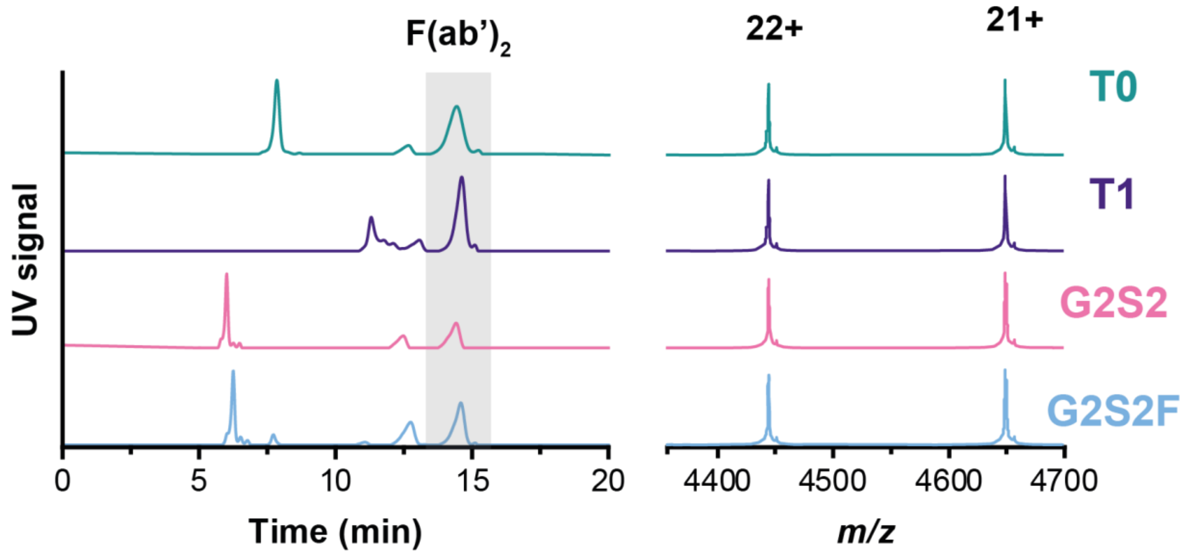

Figure S10. CEX-UV chromatogram of the different samples with a zoom of the corresponding mass spectra of the F(ab')$_2$ domain. The deglycosylation can be performed with or without the presence of a fucosidase resulting in different starting points for the transglycosylation (either GlcNAcFuc or GlcNAc).

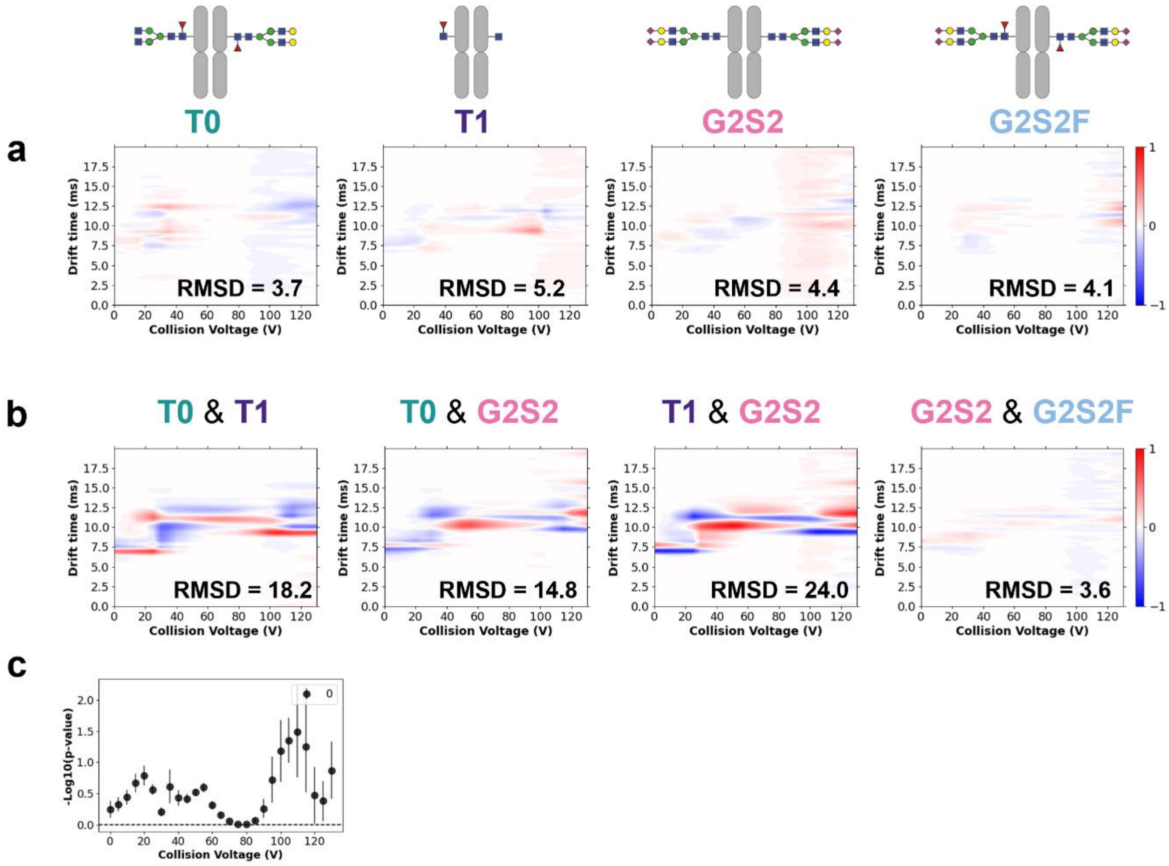

Figure S11. (**a**) RMSD plots of the replicates of the different glycoform samples showing high similarities with RSDM values below 10. (**b**) Differences between the reaction products are shown in the RMSD plots, including RMSD values. All samples showed substantial differences in CIU fingerprint, except for the G2S2 and G2S2F glycoforms. (**c**) UFS plot with the diagnostic CIU region above 100 V.

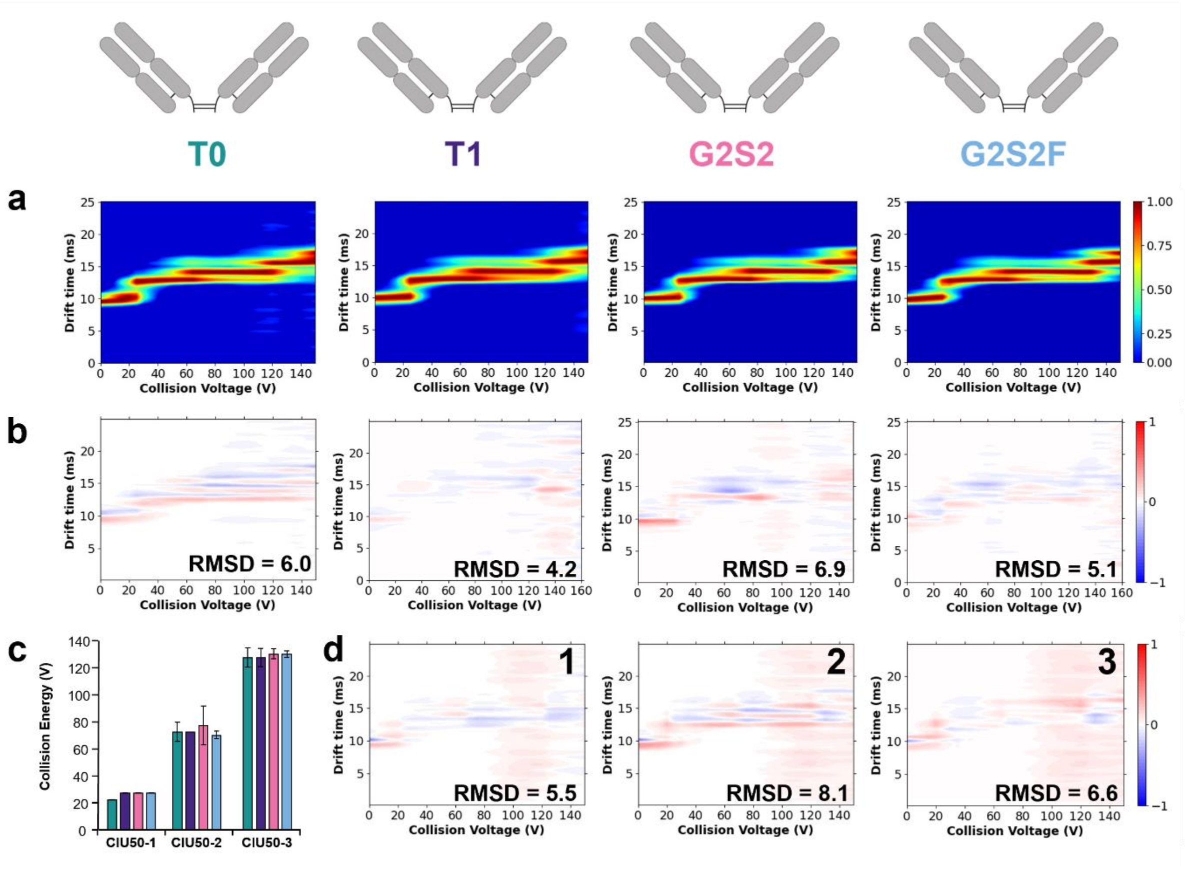

Figure S12. (**a**) CIU fingerprints of F(ab')$_2$ domain of the different trastuzumab samples, including non-treated trastuzumab (T0), deglycosylated trastuzumab (T1) and glycan-remodeled trastuzumab (G2S2 and G2S2F). (**b**) Replicate RMSD plots of the different samples, including the RMSD value. (**c**) CIU50 analysis of the F(ab')$_2$ domains of the measured samples. (**d**) RMSD plots between T0 and T1 (indicated with 1), T0 and G2S2 (indicated with 2), and T0 and G2S2F (indicated with 3) showing higher similarities of the CIU fingerprints for all samples.

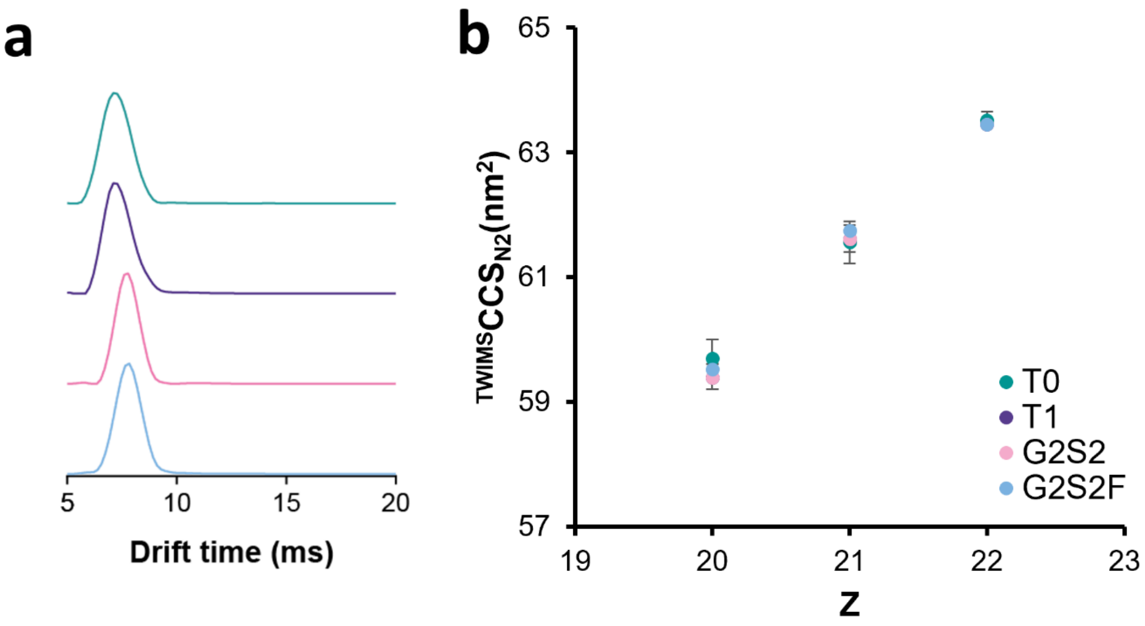

c

	T0	T1	G2S2	G2S2F
**(z)**	$^{TW}CCS_{N2}$ (nm^2)	$^{TW}CCS_{N2}$ (nm^2)	$^{TW}CCS_{N2}$ (nm^2)	$^{TW}CCS_{N2}$ (nm^2)
**20**	59.7 ± 0.3	59.5 ± 0.0	59.4 ± 0.2	59.5 ± 0.0
**21**	61.6 ± 0.3	61.7 ± 0.0	61.6 ± 0.2	61.7 ± 0.0
**22**	63.5 ± 0.1	63.5 ± 0.0	63.5 ± 0.0	63.5 ± 0.0

Figure S13. Middle-level IM−nMS analysis of Fc domains with differences in glycosylation, including untreated trastuzumab (T0, green), deglycosylated trastuzumab (T1, purple), and the glycan-remodeled trastuzumab (G2S2, pink and G2S2F, blue). (**a**) The ATDs of the 14+ charge state of the Fc fragment. (**b**) The calculated $^{TW}CCS_{N2}$ values of the Fc domains as a function of charge state. (**c**) Table summarizing the measured $^{TW}CCS_{N2}$ values.

Table S1. Retention time (RT), area, and full width at half maximum (FWHM) of intact and middle-level trastuzumab proteoforms. The CEX separation of intact trastuzumab was obtained using a gradient increasing from 50% to 70% B in 10 min. The middle-level separation was achieved by increasing the percentage of mobile phase B from 30 to 70% in 10 min. The UV chromatograms were recorded at 280 nm.

Sample	Peak	RT (min)	Area	FWHM	Resolution
Intact	Acetic	11.9	45302	0.48	1.6
	Main	13.6	290230	0.77	-
	Basic	14.9	34603	0.60	1.2
Middle-up	Fc	8.0	139510	0.32	6.6
	Fab – acetic	12.3	33805	0.52	1.5
	Fab – main	13.9	196379	0.74	-
	Fab - basic	14.8	5016	0.18	1.2

Table S2. Characteristics of the measured mAbs (eculizumab, pembrolizumab, and trastuzumab). The provided MW consists of the amino acid backbone with the G0F glycans attached. The isoelectric points (pIs) were obtained from Goyon et al. [1]. The CEX retention times (RT) were obtained using a gradient starting at 0% B for 1 min followed by a linear increase from 30% to 70% B in 9 min.

mAb	Isotype	MW (Da)	pI	RT (min)	Area	FWHM
Eculizumab	IgG2/4	147871	6.1	1.8	120194	0.30
Pembrolizumab	IgG4	148889	7.6	8.7	154065	0.27
Trastuzumab	IgG1	148056	9.1	15.1	126448	0.35

References

1. Goyon, A.; Excoffier, M.; Janin-Bussat, M. C.; Bobaly, B.; Fekete, S.; Guillarme, D.; Beck, A., Determination of isoelectric points and relative charge variants of 23 therapeutic monoclonal antibodies. *J Chromatogr B Analyt Technol Biomed Life Sci* **2017,** *1065-1066*, 119-128.
